# Pharmacological Inhibition of the PI3K/AKT/mTOR Pathway in Rheumatoid Arthritis Synoviocytes: A Systematic Review and Meta-Analysis (Preclinical)

**DOI:** 10.3390/ph18081152

**Published:** 2025-08-02

**Authors:** Tatiana Bobkova, Artem Bobkov, Yang Li

**Affiliations:** 1 Department of Rheumatology and Immunology, The Second Affiliated Hospital of Harbin Medical University, Harbin 150001, China; tat.voropaeva.1993@mail.ru; 2 National Key Laboratory of Frigid Zone Cardiovascular Diseases, The Key Laboratory of Myocardial Ischemia, Chinese Ministry of Education, Harbin 150001, China; 3 School of Health Management, Harbin Medical University, Harbin 150081, China; bobkov.a.m@yandex.com; 4 Department of Rheumatology and Immunology, Guangdong Provincial People’s Hospital (Guangdong Academy of Medical Sciences), Southern Medical University, Guangzhou 510000, China

**Keywords:** rheumatoid arthritis, fibroblast-like synoviocytes, PI3K/AKT/mTOR, pathway inhibition, p-AKT, p-mTOR, systematic review, meta-analysis, cytokines, in vitro model

## Abstract

**Background/Objectives**: Constitutive activation of the PI3K/AKT/mTOR signaling cascade underlies the aggressive phenotype of fibroblast-like synoviocytes (FLSs) in rheumatoid arthritis (RA); however, a quantitative synthesis of in vitro data on pathway inhibition remains lacking. This systematic review and meta-analysis aimed to (i) aggregate standardized effects of pathway inhibitors on proliferation, apoptosis, migration/invasion, IL-6/IL-8 secretion, p-AKT, and LC3; (ii) assess heterogeneity and identify key moderators of variability, including stimulus type, cell source, and inhibitor class. **Methods**: PubMed, Europe PMC, and the Cochrane Library were searched up to 18 May 2025 (PROSPERO CRD420251058185). Twenty of 2684 screened records met eligibility. Two reviewers independently extracted data and assessed study quality with SciRAP. Standardized mean differences (Hedges g) were pooled using a Sidik–Jonkman random-effects model with Hartung–Knapp confidence intervals. Heterogeneity (τ^2^, I^2^), 95% prediction intervals, and meta-regression by cell type were calculated; robustness was tested with REML-HK, leave-one-out, and Baujat diagnostics. **Results**: PI3K/AKT/mTOR inhibition markedly reduced proliferation (to –5.1 SD), IL-6 (–11.1 SD), and IL-8 (–6.5 SD) while increasing apoptosis (+2.7 SD). Fourteen of seventeen outcome clusters showed large effects (|g| ≥ 0.8), with low–moderate heterogeneity (I^2^ ≤ 35% in 11 clusters). Prediction intervals crossed zero only in small k-groups; sensitivity analyses shifted pooled estimates by ≤0.05 SD. p-AKT and p-mTOR consistently reflected functional changes and emerged as reliable pharmacodynamic markers. **Conclusions**: Targeted blockade of PI3K/AKT/mTOR robustly suppresses the proliferative and inflammatory phenotype of RA-FLSs, reaffirming this axis as a therapeutic target. The stability of estimates across multiple analytic scenarios enhances confidence in these findings and highlights p-AKT and p-mTOR as translational response markers. The present synthesis provides a quantitative basis for personalized dual-PI3K/mTOR strategies and supports the adoption of standardized long-term preclinical protocols.

## 1. Introduction

From the perspective of experimental modeling, the most consequential alterations in the functional properties of fibroblast-like synoviocytes (FLSs) in rheumatoid arthritis (RA) are closely linked to the dysregulation of the PI3K/Akt/mTOR signaling pathway. Under physiological conditions, this cascade sustains the homeostasis of the synoviocyte population and orchestrates the synthesis of key components of the joint microenvironment through the balanced interplay of activating and inhibitory regulators such as PTEN and TSC1/2 [1,2]. In RA, however, constitutive activation of the pathway emerges: AIM2 overexpression, disruption of regulatory protein balance (LAMTOR2/3/5, SLC38A9), and the upregulation of GLUT1, HK2, and PFKFB3 collectively drive FLSs toward an anabolic and aggressively metabolic state [3,4]. Thus, this pathway represents a promising pharmacological target and serves as a pivotal platform for translating experimental findings into clinical practice.

Nevertheless, despite substantial advances, the accumulation of data, and the emergence of large-scale integrative studies [5,6], the available evidence remains methodologically heterogeneous: variability persists in cell sources (primary RA-FLSs, HFLS-RA, MH7A), stimulation parameters (TNF-α, IL-1β, LPS), dosage ranges, and analytical time points. The evaluated metrics encompass a broad spectrum of functional and molecular endpoints—from CCK-8 assays to p-AKT levels assessed by Western blot—yet standardization and comprehensive statistical reporting are often limited to mean values ± SEM, resulting in an underestimation of variance. Collectively, these factors substantially undermine the translational reliability and reproducibility of the findings. Although recent omics preprints and studies published in 2024–2025 [7,8] have begun to provide a systems-level perspective on the signaling cascades of RA synovial tissue, a quantitative pooled synthesis of effect sizes—essential for assessing reproducibility and identifying robust therapeutic targets—remains lacking. This critical gap continues to constrain the translational fidelity of current preclinical models and impedes the development of truly personalized therapeutic strategies.

**Figure 1:**
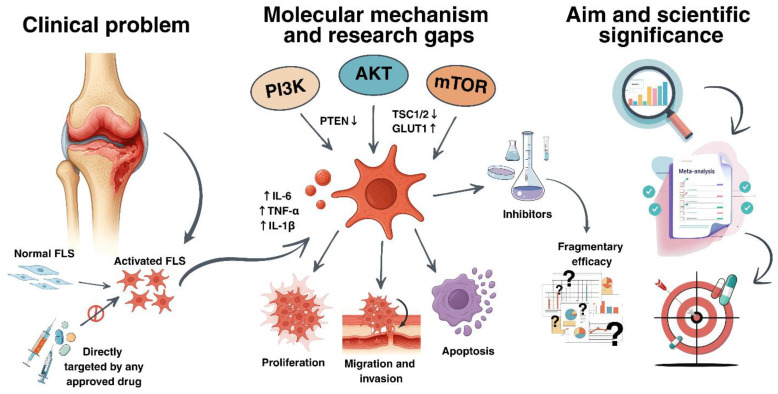
Conceptual framework: the clinical problem, key molecular mechanisms, research gaps, and the scientific rationale underpinning the necessity for a meta-analysis.

The absence of such a synthesis exacerbates the uncertainty inherent in assessing the reproducibility and comparability of observed effects, while interlaboratory variability further impedes the delineation of robust patterns and the identification of reliable molecular targets for subsequent research [9,10]. Our comprehensive search did not reveal any systematic reviews that quantitatively compared the efficacy of PI3K/AKT/mTOR inhibitors specifically in RA-FLSs, whereas isolated in vivo investigations provide only indirect support for the promise of combinatorial inhibition [11,12]. Taken together, these considerations underscore the pressing need for a rigorous and quantitative synthesis of available in vitro evidence, which is essential both for establishing well-founded scientific priorities and for identifying stable pharmacodynamic biomarkers that may serve as instruments for monitoring and individualizing therapeutic interventions in the future. A consolidated conceptual framework that integrates the clinical challenge, molecular targets, existing research gaps, and the objective of the present study is depicted in Figure 1.

The present systematic review and meta-analysis consolidates the available in vitro evidence regarding the impact of selective and combinatorial inhibition of PI3K, AKT, mTOR, as well as dual-targeted molecules, on key functional parameters of RA-FLSs—including proliferation, migration, invasion, cytokine secretion, and the phosphorylation status of pivotal signaling proteins. The primary objective of this investigation is to provide a comparative evaluation of the efficacy associated with the inhibition of distinct nodes within the PI3K/AKT/mTOR cascade, to elucidate the determinants underlying the variability of observed effects in experimental models, and to identify stable pharmacodynamic markers of inhibition suitable for monitoring therapeutic efficacy in clinical trials. It is hypothesized that pharmacological blockade of this signaling axis attenuates the pathological activity of RA-FLSs, although the magnitude of effect is expected to vary as a function of both the molecular target selected and the specificities of the in vitro protocol employed.

To achieve these objectives, the study was structured around the following aims: (i) to conduct a systematic search and critical appraisal of in vitro studies focused on PI3K/AKT/mTOR inhibition in RA-FLSs; (ii) to perform a meta-analytical quantification of the effects of these inhibitors on functional and molecular outcomes; and (iii) to investigate the impact of experimental variables on both the magnitude and reproducibility of the observed effects.

## 2. Methodology

### 2.1. Design and Registration

This systematic review and meta-analysis were conducted in full accordance with the PRISMA 2020 guidelines [13]. The protocol was prospectively developed and registered with the international PROSPERO database (ID: CRD420251058185), thereby ensuring independent documentation of methodological parameters prior to the initiation of the main search and analysis. During implementation, the publication search period was extended from the initially planned 2020–2025 interval to 2010–2025; no other amendments were made to the protocol. The scope of this review was strictly limited to experimental in vitro studies focused on the pharmacological modulation of the PI3K/AKT/mTOR signaling cascade in RA-FLSs. Particular emphasis was placed, during the meta-analytical synthesis, on molecular outcomes—including the levels of phosphorylated forms of AKT (p-AKT) and mTOR (p-mTOR)—due to their prospective translational value as reproducible pharmacodynamic biomarkers. All procedures for study identification, selection, data extraction, and statistical processing rigorously adhered to the approved protocol and are reported in accordance with the PRISMA 2020 checklist structure (see Supplementary Table S1).

### 2.2. Search Strategy

A systematic literature search was conducted exclusively in open-access bibliographic databases—PubMed (MEDLINE), Europe PMC, and the Cochrane Library—with the final search performed on May 18, 2025, without restrictions regarding language, access, or geographical location. This approach was necessitated by the limited availability of Embase, Web of Science, and Scopus at the time of the study. While this restriction may result in an incomplete coverage of the literature, the applied combination of MeSH terms and keywords—encompassing four thematic domains (pathology, cellular model, molecular targets, and type of intervention; Box 1)—alongside manual optimization and the use of the “snowballing” strategy (reference list and citation analysis in PubMed and Google Scholar), effectively minimized the risk of omitting relevant publications.

Box 1 **Example of a PubMed search query.**

("Arthritis, Rheumatoid"[MeSH] OR "rheumatoid arthritis")

AND

("Synoviocytes"[MeSH] OR "fibroblast-like synoviocyte*" OR FLS)

AND

(PI3K OR "phosphatidylinositol 3-kinase" OR AKT OR PKB OR mTOR)

AND

(inhibitor* OR antagonist* OR blockade OR modulation)

The search strategy was manually optimized through iterative variations in search terms to minimize the risk of omitting relevant publications (Supplementary Table S2), in accordance with the PRISMA-S guideline [14]. No searches were performed in gray literature sources or preprint servers (e.g., bioRxiv, medRxiv), which may impose a limitation on the comprehensiveness of the analysis due to the exclusion of non-indexed publications.

### 2.3. Inclusion and Exclusion Criteria

The selection criteria were established during the protocol development stage, based on the MICRO model, which was specifically adapted for in vitro experiments (for a detailed list of criteria, Supplementary Table S3). The review encompassed original full-text studies employing human RA-FLSs (both primary and immortalized), as well as commercially available cell lines (HFLS-RA, MH7A). Eligible publications evaluated the effects of pharmacological inhibitors of the PI3K/AKT/mTOR pathway or their dual forms, provided that a clearly defined control group and quantitative assessment of at least one relevant outcome (such as proliferation, migration, cytokine levels, or the expression/phosphorylation of signaling proteins) were present. The minimal quality criterion required either the execution of at least three biological replicates or the analysis of samples obtained from three independent donors. Studies utilizing non-arthritic or animal-derived cells, lacking pharmacological intervention, control groups, or quantitative data, as well as reviews, conference abstracts, duplicates, and publications without access to the full text, were excluded from consideration.

### 2.4. Study Selection, Screening, and Data Extraction

**Figure 2:**
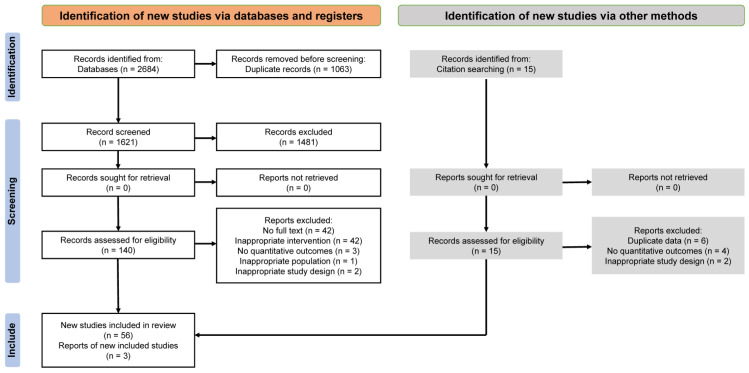
PRISMA flow diagram showing the process of literature identification, screening, eligibility assessment, and inclusion in the systematic review and meta-analysis. Numbers in each box indicate the number of publications at each stage.

The systematic search implemented in accordance with the predefined strategy identified 2684 unique publications (Supplementary Table S4). Following the removal of duplicates and the primary screening based on titles and abstracts, 140 studies were deemed eligible for full-text analysis. Qualitative assessment was conducted on 56 studies, while the final synthesis was performed on the basis of 20 (17 + 3) investigations that fully met the inclusion criteria; all stages of study selection are detailed in the PRISMA flow diagram (Figure 2). Comprehensive lists of included and excluded publications, together with the reasons for exclusion, are provided in Supplementary Tables S5–S9.

The screening procedure and data extraction were performed manually by two independent reviewers utilizing Microsoft Excel (v. 2108, Build 14332.20721); inter-rater agreement was assessed using Cohen’s kappa coefficient (κ = 0.92), and any discrepancies were resolved either by consulting a third expert or through discussion. In cases where data were available exclusively in graphical form, digitization was carried out using WebPlotDigitizer (v. 4.8; n = 88 plots; ICC = 0.93) followed by recalculation according to established formulas. All extracted parameters underwent cross-verification for completeness, consistency, and suitability for meta-analysis. A standardized extraction template was employed for the final data collection, recording details pertaining to authorship, cellular model, stimulation variants, characteristics of the investigated compounds, analyzed outcomes, and quantitative data (Supplementary Tables S10 and S11).

### 2.5. Quality Assessment of Included Studies

The methodological reliability of the included in vitro studies was evaluated using the SciRAP in vitro checklist (Supplementary Table S12A) [15]. Each study was assessed independently by two reviewers, with any discrepancies resolved through consensus. All SciRAP criteria were evaluated for each study (with percentage scores calculated for each domain), and both an overall score and a reliability category (ranging from 1 to 4) were assigned. The detailed breakdown of criteria and the corresponding heat-map matrix are provided in Supplementary Table S12B. Publications characterized by low reporting and/or methodological quality (classified as “Low” by the SciRAP framework) were not excluded from the main analysis; however, the interpretation of their results was undertaken with due consideration of the potential for high risk of bias.

### 2.6. Statistical Analysis

To mitigate biological heterogeneity, the initial meta-analysis was performed by separately aggregating comparable outcomes: functional parameters, molecular and biochemical markers, and according to the type of stimulator, as well as with consideration for the specific cellular models employed (RA-FLSs and MH7A). At this analytical stage, the concentration of stimulators was treated as a fixed variable within each group and was not regarded as an independent modifying factor.

The effect size was quantified using the standardized mean difference (SMD, Hedges’ g, 95% CI), thereby ensuring the comparability of results irrespective of the measurement scales applied. In instances where data were reported as “mean ± SEM,” the standard deviation was calculated using the formula SD = SEM × √n, where n denotes the group size [16].

The primary random-effects model employed the Sidik–Jonkman estimator for between-study variance and Hartung–Knapp (HKSJ) confidence intervals. Heterogeneity was characterized by τ^2^ (with a 95% CI based on the profile-likelihood method), Q and I^2^ statistics, and an additional 95% prediction interval (PI) was computed. The robustness of the findings was examined using a sensitivity model with REML-HK. The influence of individual studies was assessed via leave-one-out and Baujat diagnostics.

All results are detailed in Supplementary Table S13A–H. Assessment of publication bias using funnel plots and statistical tests (Begg’s, Egger’s) was not performed, as the number of studies included for all aggregated outcomes was fewer than five (k < 5); accordingly, the analysis was conducted solely in a qualitative manner, with careful consideration of the general context and the potential impact of publication bias in the interpretation of results. For additional verification of the stability of the detected effects, univariable meta-regression analyses by cell type were performed (Supplementary Table S14). The certainty of the evidence was not assessed due to the in vitro design of all included studies and the inapplicability of the GRADE methodology.

All calculations and visualizations were performed in VSCode v.1.100.3 (Python 3.12, R 4.5) utilizing specialized packages; the original scripts and analytical pipeline are provided in Supplementary Files S16–S18.

## 3. Results

### 3.1. Risk of Bias Assessment (RoB) Using the SciRAP Tool

Assessment of the included studies using the SciRAP checklist revealed a mean Reporting Quality score of 70.3% (range: 22.4–77.6%) and a Methodological Quality score of 71.5% (42.5–77.5%). The majority of studies were classified as “moderate quality” (n = 18, 90%), with only one study each categorized as “low quality” (n = 1, 5%) and “high quality” (n = 1, 5%). The most frequent limitations pertained to insufficient specification of reagent purity, lack of positive controls, and incomplete reporting of measures taken to prevent contamination of cell cultures. Consequently, while the overall reliability of evidence is considered satisfactory, the identified limitations—particularly in studies of low and moderate quality—necessitate cautious interpretation of large effect sizes (|g| > 2.0). The full quality assessment table is presented in Supplementary Table S12C.

### 3.2. Quantitative Synthesis of Aggregated Effects

#### 3.2.1. Phenotypic Effects of PI3K/AKT/mTOR Inhibitors

**Figure 3:**
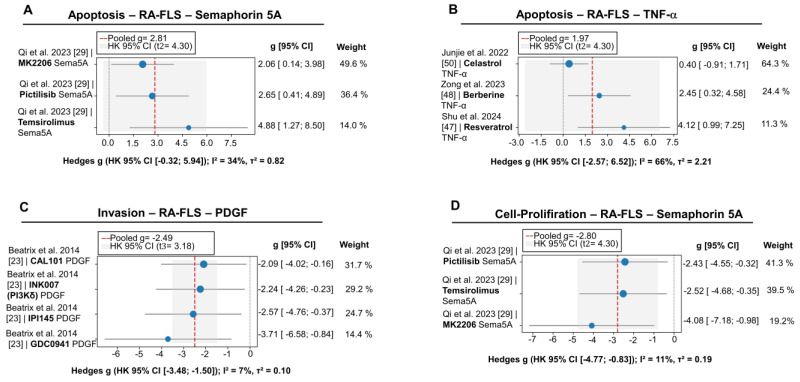

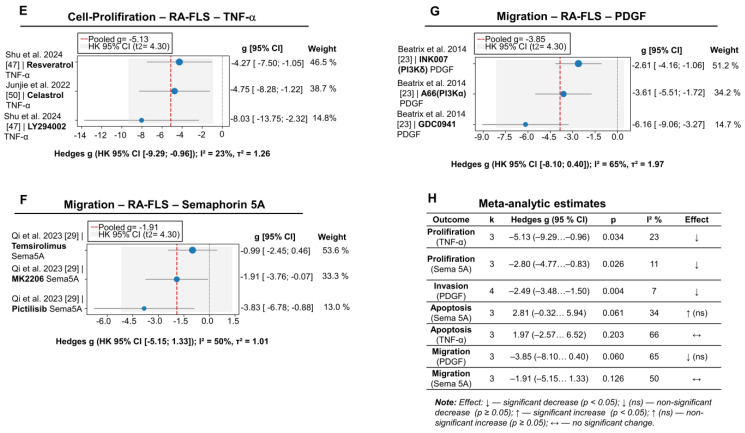
The impact of PI3K/AKT/mTOR inhibitors on the phenotypic characteristics of RA-FLSs. (**A**–**G**) Forest plots illustrating the effects of pharmacological inhibition of the PI3K/AKT/mTOR signaling pathway on the following phenotypic outcomes: apoptosis under stimulation with Semaphorin-5A (**A**) and TNF-α (**B**); invasiveness under PDGF stimulation (**C**); proliferative activity under Semaphorin-5A (**D**) and TNF-α (**E**); migratory potential under stimulation with Semaphorin-5A (**F**) and PDGF (**G**). Effect sizes are presented as standardized mean differences (Hedges’ g) with 95% confidence intervals (CI). (**H**) Summary table of integrated meta-analytic estimates for all phenotypic outcomes, indicating the direction and magnitude of the effect as well as statistical significance. The ranges presented reflect the minimum and maximum observed changes for each phenotypic parameter among all included studies. Assessment of the certainty of evidence was not performed.

**Figure 4:**
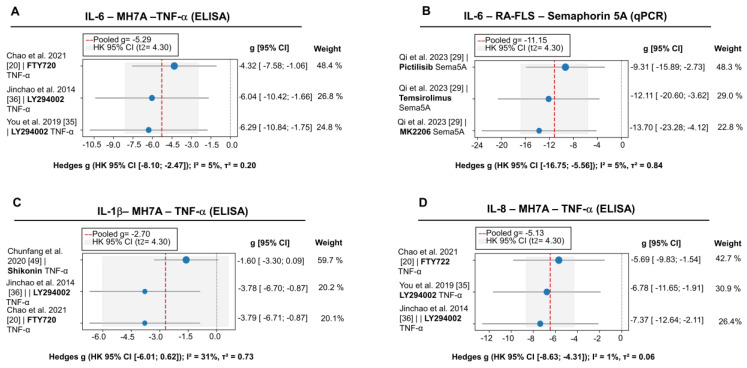

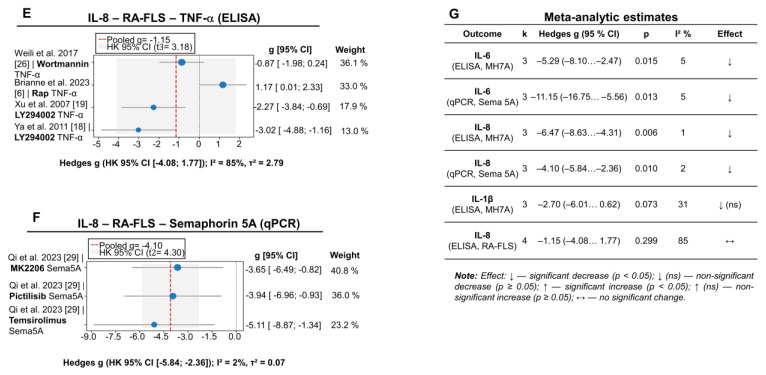
Effects of PI3K/AKT/mTOR inhibitors on the production of proinflammatory cytokines in RA-FLSs. (**A**–**F**) Forest plots depicting changes in the production of IL-6 (**A**,**B**), IL-1β (**C**), and IL-8 (**D**–**F**) in response to pharmacological inhibition of the PI3K/AKT/mTOR signaling cascade under various stimuli and cellular models (primary RA-FLSs and the MH7A cell line). Standardized mean differences (Hedges’ g) with 95% confidence intervals (CI) are presented. (**G**) Summary table of meta-analytic estimates, consolidating the magnitude and direction of effects for each cytokine, as well as statistical significance and heterogeneity.

The conducted meta-analysis demonstrates that inhibition of the PI3K/AKT/mTOR signaling cascade is associated with a marked suppression of the proliferative activity of RA-FLSs, and these effects are consistently reproducible regardless of the stimulus employed—be it Semaphorin-5A or TNF-α (g ranging from –2.80 to –5.13; see Figure 3H). In the PDGF stimulation model, the reduction in invasiveness reaches a clinically meaningful magnitude (g = –2.49; Figure 4C). In contrast, the dynamics of apoptotic changes are limited to a trend toward enhancement following Semaphorin-5A exposure (g = 2.81; *p* = 0.061; Figure 3A), while the TNF-α context fails to reach statistical significance, accompanied by pronounced inter-study heterogeneity (I^2^ = 66%; τ^2^ = 2.21; Figure 3B) and a 95% prediction interval crossing the null line (PI: –5.9 to 9.8). The migratory potential of the cells likewise exhibits ambiguity: a borderline decrease is observed for PDGF (g = –3.85; *p* = 0.06; Figure 3G), whereas Semaphorin-5A exerts no statistically significant influence (Figure 3F). Sensitivity analyses confirmed the robustness of the observed associations.

#### 3.2.2. Effects on the Production of Proinflammatory Cytokines

Pharmacological blockade of the cascade is accompanied by a substantial and consistently reproducible reduction in the production of IL-6 and IL-8—both at the level of secretion in MH7A cells (g = –5.29 and –6.47; see Figure 4A,D) and in terms of expression in primary RA-FLSs activated by Semaphorin-5A (g = –11.15 and –4.10; Figure 4B,F). In contrast, the decrease in IL-1β secretion is limited to a non-significant trend (g = –2.70; *p* = 0.073; Figure 4C), while the pronounced inter-study variability in the IL-8 response under TNF-α stimulation (I^2^ = 85%) precludes the attainment of statistical significance (g = –1.15; *p* = 0.299; Figure 4E).

#### 3.2.3. Alterations in PI3K/AKT/mTOR Signaling Cascade Activity

**Figure 5:**
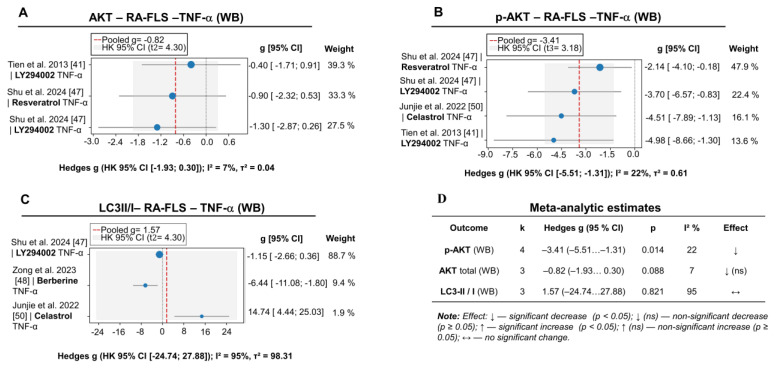
The impact of PI3K/AKT/mTOR pathway inhibition on intracellular markers of cascade activity and autophagy. (**A**–**C**) Forest plots illustrating changes in the total AKT level (**A**), the phosphorylated form of AKT (p-AKT) (**B**), and the integral autophagy marker LC3-II/I (**C**) following pharmacological inhibition of PI3K/AKT/mTOR. Effect sizes are presented as standardized mean differences (Hedges’ g) with 95% confidence intervals (CI). (**D**) Summary table of meta-analytic estimates, synthesizing the direction and significance of changes in intracellular signaling markers and autophagy markers.

Building upon the alterations identified at the phenotypic and cytokine levels, the analysis of intracellular markers reveals that PI3K/AKT/mTOR inhibition induces a coherent reduction in phosphorylated AKT (g = –3.41; 95% CI: –5.51 to –1.31; *p* = 0.014; see Figure 5B), reflecting the efficacy of pathway blockade and aligning with the observed anti-proliferative and anti-invasive effects. Additional sensitivity analyses corroborated the robustness of the pAKT effect (g = –4.29; 95% CI: –5.92 to –2.66), thereby strengthening confidence in the overall conclusions.

Conversely, the total AKT level exhibited only a marginal decrease, bordering on statistical significance (g = –0.82; *p* = 0.088; Figure 5A), which may suggest a predominant modulation of kinase activity rather than total protein expression. The LC3-II/I ratio, an integral marker of autophagy, did not undergo significant changes (g = 1.57; *p* = 0.821; Figure 5C), although pronounced inter-study heterogeneity (I^2^ = 95%) complicates the unequivocal interpretation of this result.

#### 3.2.4. Meta-Regression Analysis of the Impact of Cell Type on Effect Size

An additional meta-regression analysis was undertaken to delineate the influence of cell type on the magnitude of inhibitor effects, employing a categorical moderator variable distinguishing between primary RA-FLSs and the immortalized MH7A cell line. Owing to insufficient variability or the limited volume of data for other biomarkers, the analysis was feasible only for two outcomes—IL-6 and IL-8 production (see Table 1 and Table S14).

**Figure 6:**
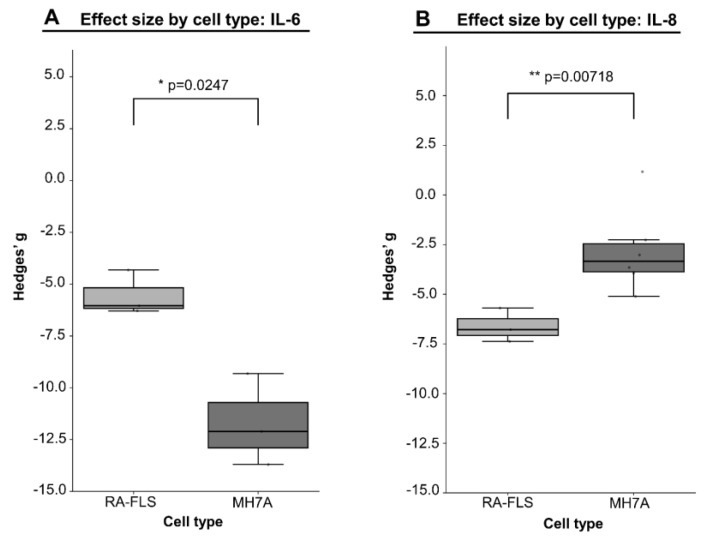
Impact of cell type on the effect size of PI3K/AKT/mTOR inhibitors (Hedges’ g) with respect to IL-6 and IL-8 production. The plot presents individual effect size values (dots), medians, interquartile ranges, and outliers for each cell type. Each point represents a distinct study; the boxplot displays the median, quartiles, and outliers for RA-FLS and MH7A cell types.

For IL-6 production, the moderator exhibited a pronounced negative effect (b = –5.85; *p* = 0.025), formally indicating a more substantial suppression of this cytokine in primary RA-FLSs compared to the immortalized MH7A line. Conversely, for IL-8, the relationship was inverted: a significant positive effect (b = 4.10; *p* = 0.037) denotes a more pronounced inhibition of IL-8 production specifically in MH7A cells. Visualization of these findings through boxplot graphs (Figure 6) enables a clear depiction of the described intercellular differences. Notably, the median effect size for IL-6 in primary RA-FLSs reaches –12, nearly twice that observed for MH7A (approximately –6). In contrast, for IL-8, the most pronounced and consistent effect is evident in the MH7A cell line (around –7, versus –3 in RA-FLSs).

Thus, the observed variability in effects depending on cell type underscores the importance of accounting for cellular context when interpreting experimental data and planning further investigations. At the same time, the limited data for other outcomes highlights the need for standardization of experimental models and for expanding the spectrum of cell lines in order to achieve a comprehensive evaluation of inhibitor efficacy.

### 3.3. Qualitative Synthesis: Classification and Mechanisms of Interventions Targeting the PI3K/AKT/mTOR Axis

#### 3.3.1. General Characteristics of Included Studies

**Figure 7:**
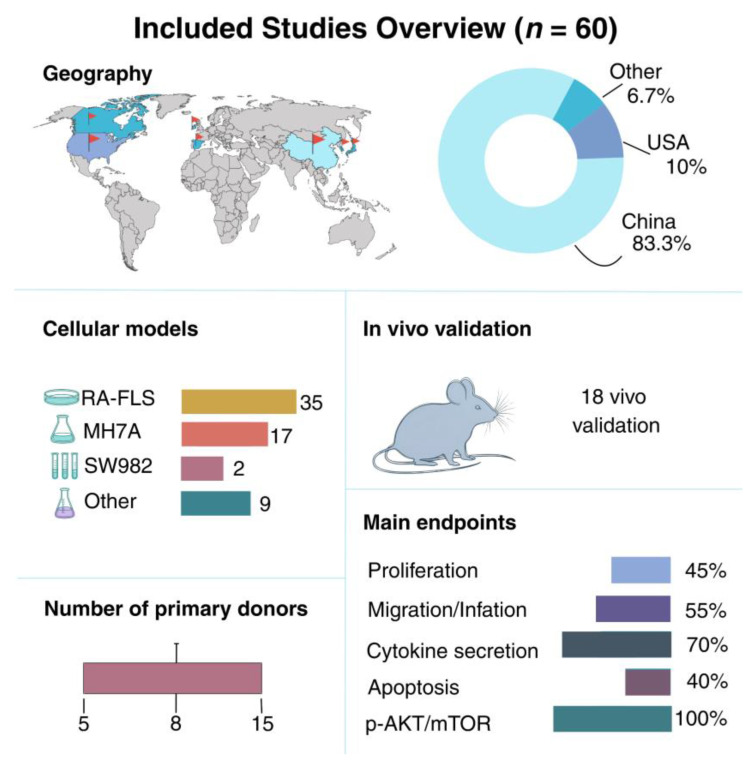
Profile of studies included in the qualitative synthesis: geographical distribution, cell models utilized, number of primary donors, principal experimental endpoints, and frequency of in vivo validation.

The summary infographic (Figure 7) illustrates the distribution of source countries, cell models, metrics employed, and other parameters of the studies included in the analysis.

The classification of interventions and the principal effects of different compound groups on RA-FLSs are presented in Table 2.

#### 3.3.2. Classical Pharmacological Agents and Drug Repurposing (n = 7)

In the context of translating preclinical findings into clinical therapies for RA, considerable attention has been drawn to pharmacological modulators of the PI3K/AKT/mTOR cascade that impact the functional profile of RA-FLSs.

A series of original investigations has demonstrated that exposure to rapamycin (100 nM, 24 h) in TNF-α-induced cells not only induces a sustained reduction in phosphorylated S6 levels, but is also accompanied by suppression of glycolytic activity, a decrease in reactive oxygen species production, and a marked reduction in the expression of cell adhesion molecules [6]. Paclitaxel (2.5–10 nM, 24–48 h), though distinct in its mechanism of action, elicits a comparable effect, manifested by the inhibition of RA-FLS migration (up to 70%), diminished cytokine and metalloproteinase expression, yet with negligible induction of apoptosis, indicative of a predominantly cytostatic mechanism [17]. Artesunate (0.5–20 μM, 24 h) has been shown to substantially decrease VEGF, IL-8, p-Akt, and HIF-1α levels, collectively resulting in a 20–45% reduction in RA-FLS viability [18,19]. Similarly, it has been demonstrated that the S1P receptor modulator fingolimod (FTY720), when administered orally to DBA/1J mice at 2 mg/kg daily for 35 days, markedly suppressed CD4^+^ T-lymphocyte recruitment to the joints in a collagen-induced arthritis model, while in MH7A cell cultures, it inhibited TNF-α-induced activation of PI3K/Akt/NF-κB and reduced secretion of IL-1β, IL-6, and IL-8 [20]. Notably, these agents, irrespective of molecular nature, are capable of significantly inhibiting migratory and invasive activity—by up to 70% in certain experimental series.

These findings reflect an increasing appreciation that modulation of signaling pathways exerts influence over a broad spectrum of cellular functions; nevertheless, interpretation of the results requires a degree of circumspection. It must be emphasized that the majority of the reported data were obtained under short-term in vitro models employing a limited repertoire of cell lines, frequently immortalized or primary, which inevitably restricts their translatability to clinical practice. Another critical aspect is the lack of adequate donor stratification and the small size of the cohorts examined, which complicates the verification of effect durability and diminishes the level of reproducibility [6,21]. Such a limitation profile necessitates further investigations in expanded and stratified samples, with particular emphasis on long-term functional outcomes. The ranges of observed effects are summarized in Table 3.

These findings reflect an increasingly nuanced understanding that modulation of signaling pathways exerts multifaceted effects on cellular function; nevertheless, a degree of caution in the interpretation of these results remains warranted. It is essential to underscore that the majority of data presented were derived from short-term in vitro models utilizing a limited range of cell lines—frequently immortalized or primary—which unavoidably constrains their translatability to clinical practice. Another critical limitation lies in the insufficient stratification by donor and the small size of the studied cohorts, both of which hinder verification of effect stability and undermine reproducibility [6,21,22]. Notably, while pronounced reductions in arthritis index were observed in in vivo models, the absence of experiments employing human RA-FLSs and the limited characterization of donor profiles markedly restrict the extrapolation of these results. This constellation of limitations underscores the necessity for future research employing larger, stratified cohorts with a specific focus on long-term functional outcomes.

#### 3.3.3. Targeted Small-Molecule Inhibitors (n = 17)

From the perspective of clinical translation, targeted small-molecule inhibitors of the PI3K/AKT/mTOR cascade merit particular attention, as they afford highly selective modulation of RA-FLS activity while minimizing off-target effects and expanding the therapeutic window.

A number of studies have demonstrated that PI3Kδ inhibitors (INK007, IPI-145, CAL-101) possess pronounced translational potential—capable of attenuating migration, invasion, and cytoskeletal remodeling of RA-FLSs by 60–85% under PDGF-BB or TNF-α stimulation, concomitant with blockade of AKT, Rac1, and PAK phosphorylation [23,24]. Nevertheless, these effects are often reproduced in a limited array of cell lines, frequently without donor stratification or comprehensive validation of functional robustness. The substantial impact of macrophage-derived factors and angiogenic stimuli is evident in the induction of vimentin and type II collagen expression, as well as the activation of Erk1/2, JNK, and AKT pathways—findings corroborated by interventions with PDGF-BB and PI3K/ERK inhibitors [25,26]. Of note is the dual inhibitor NVP-BEZ235, which elicits a rapid reduction in RA-FLS proliferation through blockade of the PI3K/mTOR axis, as well as combined regimens with IL-22 that suppress pathological activation [27].

It should be emphasized that the characteristics of donor material and the number of independent replicates are often undisclosed, thereby limiting reproducibility and generalizability. In several instances, there is a lack of evaluation of long-term effects and the durability of outcomes. While efficacy against alternative targets such as eEF2K (NH125) and Semaphorin 5A illustrates the breadth of therapeutic possibilities—manifested by suppressed RA-FLS metabolism and reversible attenuation of inflammation in model systems [28,29]—the paucity of data on biomaterial variability and remission parameters precludes unequivocal conclusions regarding the translatability of these effects. The observed ranges of effects for the targeted small-molecule inhibitor group are systematically summarized in Table 4.

In this context, interventions aimed at epigenetic and multi-targeted signaling modulation are of particular interest, as they enable the “reprogramming” of entrenched activation patterns in RA-FLSs and suppression of elements within the inflammatory cascade. Thus, restoration of PTEN expression via 5-azadC or genetic overexpression (Ad-PTEN) resulted in a 45–60% reduction in p-AKT levels, suppression of proinflammatory cytokine and metalloproteinase expression, as well as a significant decrease in edema in inflammation models [30]; combined modulation of Wnt5a signaling with inhibition of PI3K, MAPK, and ROCK led to marked reductions in RA-FLS migration and production of inflammatory mediators [31]. In addition, a number of multi-purpose compounds—such as dafnitin, rhodojaponin II, genistein, diosmetin, morin, cinnamaldehyde, among others—exerted comprehensive effects on multiple regulatory axes, including PI3K/AKT/mTOR, NF-κB, and MAPK, inducing dose-dependent suppression of proliferation (−20 to −50%), inhibition of migration (up to −90%), enhancement of apoptosis, and reduction in the production of inflammatory and angiogenic mediators [32,33,34,35,36,37,38,39]. Exogenous APEX1, through its effects on PI3K and NF-κB, additionally supported mitochondrial function and mitigated the aggressive phenotype of RA-FLSs [40], while resveratrol elicited suppression of IL-1β, IL-6, MMP-3 expression, and cell proliferation via the PI3K/AKT pathway [41]. Finally, integration of combination strategies involving simultaneous inhibition of multiple signaling axes—such as PI3K/mTOR, MAPK, and AMPK—demonstrates the potential for further enhancement of anti-pathogenic efficacy, thereby establishing a novel framework for subsequent preclinical and clinical validation [25,38].

Critically, for the majority of these studies, limitations also include reliance on a single cell line (e.g., SW982, MH7A), the absence of primary RA-FLSs or donor heterogeneity, a small number of replicates (n = 3–6), lack of randomization and long-term in vivo corroboration, as well as frequently incomplete statistical reporting and omission of negative or vehicle control groups.

#### 3.3.4. Phytochemical Agents and TCM Formulations (n = 20)

Transitioning from molecularly targeted strategies to natural and traditional modalities, the contemporary research agenda places increasing emphasis on phytochemical agents and multi-component formulations derived from traditional Chinese medicine (TCM), which have the potential to exert a multifaceted influence on the pathological phenotype of RA-FLSs.

Among the most extensively studied compounds: Paederia scandens extract demonstrated a 20–57% reduction in cell viability and an increase in apoptosis up to 79%, accompanied by pronounced suppression of IL-1β, IL-6, and IL-17 production [42]; tanshinone IIA achieved inhibition of IL-6, IL-8, and MMP-9 by 70–85%, and in AIA animal models led to a 40–50% reduction in joint edema [43]. In other cases, astragaloside IV induced cell cycle arrest and reduced p-AKT by 45–60%, as corroborated by rescue experiments with lncRNA LOC100912373 expression [44], while cinnamaldehyde in a rat model reduced joint edema by 45–50% and tripled the frequency of apoptosis [34]. Additionally, naringin and diosmetin suppress inflammatory and invasive RA-FLS activity via inhibition of the PI3K/Akt and MAPK/ERK pathways, reducing cytokine and matrix metalloproteinase production and enhancing apoptosis [35,45], while genistein further modulates ROS/Akt/NF-κB signaling [36]. Artemisitene (ATT) also warrants consideration, as it has demonstrated pronounced reduction in the proliferation and induction of apoptosis in RA-FLSs through suppression of the PI3K/AKT axis and m6A regulation [46]. Similarly, berberine exhibits the ability to inhibit autophagy and induce apoptosis in RA-FLSs via regulation of the ROS-mTOR axis; resveratrol, by blocking PI3K/Akt signaling and reducing cytokine secretion, manifests both antiproliferative and anti-inflammatory effects [47,48], while artesunate suppresses IL-1β, IL-6, and IL-8 production, acting primarily through inhibition of NF-κB and PI3K/Akt pathways [19]. The ranges of changes for the principal cellular effects are summarized in Table 5.

Nonetheless, the encouraging effects of most phytochemical agents and TCM formulations are accompanied by typical methodological limitations: a predominance of studies utilizing a single cell line or animal model [39,49,50], employment of pharmacologically elevated concentrations, and a lack of transparent standardization of extract composition (quality standards were clearly declared in only a minority of studies).

Against the backdrop of the aforementioned methodological limitations, the question arises as to how profoundly and comprehensively phytochemical agents are capable of modulating intracellular metabolism and signaling axes in RA-FLSs. It is precisely in this context that the diversity of mechanisms and the rationale for their inclusion in contemporary experimental therapeutic regimens become evident. For example, shikonin reduced ATP levels to 35–41% and markedly enhanced autophagy in RA-FLSs, while concurrently diminishing clinical manifestations of arthritis in rats, with no changes observed in indices of hepatotoxicity [49,51]. Rhodojaponin II and dafnitin likewise exhibited pronounced inhibition of PI3K/AKT/mTOR signaling, suppressing proliferation, migration, and invasion of cells [32,33]. Baicalin and myricetin were characterized by their ability to inhibit EMT, reduce expression of PI3K/Akt/mTOR and markers of RA-FLS aggressiveness, as well as suppress production of IL-6, IL-8, and metalloproteinases [4,52]. Comparable effects were noted for protocatechuic acid, which suppressed p-Akt/p-mTOR and promoted apoptosis [53], and for morin, which inhibited multiple signaling pathways and reduced RA-FLS migration by 50–82% [38]. In the context of antioxidant therapy, glutathione (GSH) suppressed ROS, reduced proinflammatory cytokines, and activated PTEN expression by inhibiting p-PI3K/p-AKT [54]. Decoctions and complex TCM formulations, such as JBT, have shown potential in regulating the balance of proinflammatory mediators and invasion of RA-FLSs, though they require further standardization and large-scale clinical validation.

Despite the demonstrated potential of phytochemical compounds, the majority of studies are constrained by short-term experimental designs, lack of standardization of composition, and a small number of independent observations [10,43,55,56]. Significant gaps pertain to the absence of long-term assessment, insufficient donor stratification, and low transparency in reporting the origin of raw materials, all of which impede comparability of results across laboratories. Additionally, reversibility of effects following drug withdrawal and variability in dosages employed further complicate the objective evaluation of therapeutic potential [57].

#### 3.3.5. Genetic and Epigenetic Interventions (n = 10)

Advancing from non-specific phytopreparations to molecular precision, particular attention should be devoted to genetic and epigenetic interventions. While studies in cellular and animal models consistently demonstrate a profound attenuation of inflammatory and proliferative processes, the clinical translation of these approaches remains contingent upon addressing several fundamental challenges, including the assessment of off-target effects, the determination of long-term safety, and the development of efficacious delivery systems for therapeutic constructs.

For example, modification of m^6^A methylation of ICAM2 mRNA by artemisitene resulted in a 55–70% reduction in ICAM2 expression and p-AKT levels, accompanied by a marked decrease in proliferation and migration of RA-FLSs [46]. Epigenetic restoration of PTEN via 5-azadC or overexpression demonstrated a significant attenuation of the inflammatory phenotype through inhibition of p-AKT and proinflammatory cytokines [30]. Interventions utilizing shRNA targeting RAC2, METTL3, or pcDNA-RAC2 led to reduced proliferation, enhanced apoptosis, and regulation of ROS/SOD/MDA [58], while blockade of the SLC7A5 transporter (siRNA-SLC7A5) and modulation of long-ncRNA expression, including LOC100912373 and HOTAIR, enabled control over the cell cycle and angiogenesis via the PI3K/AKT/VEGF axis [59,60,61]. Approaches targeting autophagy and the cytoskeleton, including inhibition of METTL14 or modulation of FASN, provided combined inhibition of mTOR/ROS and decreased explant activity of RA-FLSs [62,63]. However, all these studies rarely compared their findings with established standards of therapy (DMARDs, biological agents), rendering their practical significance largely hypothetical. Moreover, for most such approaches, long-term off-target effects and the feasibility of adaptation to diverse clinical scenarios remain undefined, given the exceptional heterogeneity of the RA patient population. The spectrum of changes observed in the context of genetic and epigenetic interventions is summarized in Table 6. The ranges of changes observed with genetic and epigenetic interventions are presented in Table 6.

Particular research precision in this domain is introduced by rescue experiments—laboratory scenarios in which intervention in a given signaling pathway can not only be induced but also reversed or abrogated. Such approaches (e.g., suppression of long-ncRNA LOC100912373 expression [60]) become a veritable criterion of validity for a molecular axis. The implementation of these strategies enables the stepwise testing of involvement across various cascades and substantiates the functional significance of specific nodes [26]. For example, administration of a specific inhibitor (si-THRIL) reduced IL-1β levels by 45% and simultaneously demonstrated decreased clinical activity in the model [64], while genetic targeting of transporters and matrix components (e.g., SLC7A5, KIAA1199) resulted in decreased RA-FLS invasion and metalloproteinase secretion [29,59], and combined interventions involving microRNAs, as exemplified by miR-214 and tocilizumab, indicated the potential for integrating novel approaches into therapeutic regimens [21]. Furthermore, epigenetic interventions—such as suppression of METTL14 or lncRNA HOTAIR—ensured reduced proinflammatory cytokines and decreased joint swelling by 30–50% in the CIA model [61,62].

Nonetheless, despite their analytical sophistication, the limitations of such approaches are considerable. Firstly, the majority of rescue experiments have been conducted using a limited array of cell lines or animal models, which precludes assessment of effect heterogeneity within the actual patient population. Secondly, the emphasis on molecular readouts and short-term outcomes frequently excludes the tissue and systemic context of the disease from analysis, thereby complicating the extrapolation of results. Moreover, the high specificity of intervention does not preclude the possibility of cross-influence on ancillary signaling axes and compensatory mechanisms, which may distort the true role of the target under investigation. Collectively, this demonstrates that, despite the fundamental value of rescue experiments for the mapping of pathogenetic pathways, their clinical relevance remains a matter of debate and necessitates further validation in more representative models.

#### 3.3.6. Nanoplatforms, Biomaterials, and Physical Methods of Intervention (n = 5)

Whereas previous strategies predominantly emphasized systemic modulation of cellular pathways and inhibition of pivotal signaling cascades at the organismal level, there is now a decisive paradigm shift toward localized interventions—namely, targeted delivery of therapeutic agents, controlled release systems, and biomimetic modification of the joint microenvironment. The promise of nanoplatforms and innovative physical modalities lies not only in their capacity to directly suppress RA-FLS activity and local inflammation, but also in their ability to minimize the adverse effects of systemic agents, enable therapy personalization, and surmount the drug resistance that typifies chronic joint inflammation. Nevertheless, the pathway to clinical implementation of these technologies necessitates substantial refinement and the overcoming of multiple translational barriers.

Notably, multifunctional hydrogels based on hyaluronic acid and gold nanoparticles (HA-Au@RGD/triptorelin) with NIR activation enable targeted drug accumulation in inflamed tissues (resulting in a 6.4-fold increase in fluorescence signal), pronounced reduction in RA-FLS proliferation by 35–54%, and decreased p-mTOR and p-p70S6K activity by 38–54%; cytokine expression (IL-6, TNF-α) was reduced by 20–60% at one-third the dose compared to traditional regimens [65]. Nevertheless, the technological novelty, brevity of protocols, and absence of comprehensive toxicological profiling objectively hinder their translation beyond experimental models. Equally ambitious are exosomal technologies and hybrid nanoparticles (e.g., BMDM-sEVs, (Zn-Adenine)@Ab@LEF1-AS1 NP), which not only impact cellular mechanisms [66], but also effectuate a sharp reduction in RA-FLS pathogenicity and erosion rates in experimental models (clonogenicity reduced by 65–70%, erosion frequency decreased from 80% to 18%) [67]. However, the high complexity of synthesis, lack of standardization and multicenter validation, and limited supply of donor material or animal models leave questions of reproducibility and transferability unresolved.

Biomimetic nanoplatforms (e.g., HA@RFM@GP@SIN-NPs) and 3D organoids, in turn, demonstrate the potential for comprehensive macrophage repolarization, inhibition of RA-FLS migration, and reduction in therapeutic resistance by 45–60% [68], yet continue to be studied only in short-term and relatively homogeneous laboratory models. It is also important to mention physical interventions (e.g., 630 nm LED photobiomodulation), which may open a new avenue for non-invasive suppression of proinflammatory cascades, decreasing IL-6 and IL-8 by 50–60% through TRPV4 activation and PI3K/AKT/mTOR inhibition; however, the question of structural consequences and long-term safety of these interventions requires separate investigation [69]. The ranges of effects for nanoplatforms, biomaterials, and physical interventions are summarized in Table 7.

Collectively, it is technological complexity, short observation horizons, and a paucity of toxicity data that currently define the limits of clinical extrapolation: these new platforms represent not an alternative, but a strategic reserve to be drawn upon as mature, standardized protocols for preclinical and toxicological validation emerge, as well as within the context of interlaboratory and multicenter studies with extended timeframes. Absent the resolution of these challenges, innovative platforms will remain at the stage of laboratory demonstration, and their transition to clinically viable tools will be indefinitely deferred.

## 4. Discussion

### 4.1. Brief Summary of the Obtained Data

In the course of the quantitative synthesis, which encompassed 17 stratified comparisons (each comprising 3 to 4 primary experiments), a consistent attenuation of phenotypic features of RA-FLSs was observed upon pharmacological inactivation of the PI3K/AKT/mTOR cascade. Fourteen clusters exhibited a pronounced negative integral effect (|g| ≥ 0.8 SD), indicative of a substantial diminution in proliferation, proinflammatory cytokine secretion, and cellular migration/invasion; the most prominent alterations were documented for TNF-α-induced proliferation of RA-FLSs (g ≈ –5.1 SD), IL-6 expression by qPCR upon Semaphorin 5A stimulation (g ≈ –11.1 SD), and IL-8 secretion by ELISA in MH7A (+TNF-α) (g ≈ –6.5 SD). Concurrently, two apoptotic clusters (g ≈ +2.7 SD and +2.0 SD) and the LC3II/LC3I ratio (g ≈ +1.6 SD) demonstrated an increase, consonant with the activation of autophagic and apoptotic processes. In 11 out of 17 comparisons, heterogeneity was assessed as low or moderate (I^2^ ≤ 35%), while a marked escalation of heterogeneity (I^2^ > 60%) was observed solely for specific phenotypic parameters—apoptosis, IL-8 secretion, LC3II/LC3I, and migration upon PDGF stimulation; notably, in all instances, the directionality of the effect persisted irrespective of variations in dosages and experimental protocols.

The transition from the Sidik–Jonkman variance estimator to REML (both with Hartung–Knapp adjustment) in sensitivity analyses resulted in only minimal displacement of the integral estimate (mean, 0.05 SD; maximum, 0.18 SD for VEGF, qPCR). Leave-one-out analysis demonstrated that the exclusion of any individual experiment did not alter the directionality of the effect, with the largest observed shift (≈ 1.1 SD) arising from the removal of Shikonin–TNF-α for IL-1β, yet the result retained its negative orientation. Baujat diagnostics enabled the identification of the most influential experiments (Shikonin–TNF-α for IL-1β, FTY721–TNF-α for IL-6, MK2206–Sema5A for proliferation, and Temsirolimus–Sema5A for VEGF); however, their exclusion did not lead to any alteration of the principal conclusions.

Thus, the cumulative suppression of proliferation, IL-6/IL-8 production, migration/invasion, and AKT/mTOR signaling activity is characterized by high biological and methodological robustness, with the stability of results across alternative dispersion models and exclusion of potentially atypical experiments attesting to the validity of the findings regarding the anti-inflammatory potential of targeted PI3K/AKT/mTOR inhibition in RA-FLSs. Among the most promising candidates for translational application are the biomarkers p-AKT and p-mTOR, both of which exhibited remarkable stability and a clear pharmacological responsiveness. These data establish a substantive evidentiary basis for incorporating assessments of phosphorylated AKT and mTOR levels as reliable pharmacodynamic endpoints in the design of future preclinical and clinical trials. The complete set of indicators, including all sensitivity analyses, is presented in Supplementary Table S13.

### 4.2. Biological Interpretation of the Results

#### 4.2.1. Disruption of the “FLS–Macrophage” Inflammatory Circuit

A detailed analysis of the underlying biological mechanisms elucidated in this study delineates the therapeutic points of intervention within the context of rheumatoid inflammation. Specifically, inhibition of the PI3K/AKT/mTOR signaling pathway results in a simultaneous and substantial reduction in the expression of key proinflammatory cytokines, thereby disrupting the self-sustaining autocrine and paracrine circuit between fibroblast-like synoviocytes and macrophages. This autocrine–paracrine network, maintained by PI3K-dependent phosphorylation of IKKβ and c-Jun, constitutes the foundation for the chronicity of inflammation through persistent activation of the transcription factors NF-κB and AP-1 [70,71]. Proximal blockade of the PI3K/AKT cascade thus dismantles the pathological communication between RA-FLSs and M1-polarized macrophages. The significance of these findings is further reinforced by in vivo models, where a greater than 40% reduction in IL-6 levels in synovial fluid is accompanied by a shift in macrophage phenotype toward M2 and a pronounced decrease in osteoclastogenesis [72,73]. In most instances, the in vitro effects identified in our study exceed this threshold, underscoring not only the molecular soundness of this approach, but also its translational promise for the development of clinically mature strategies for targeted therapy.

#### 4.2.2. Cellular Phenotype: Proliferation, Apoptosis, and Pannus Regression

Elaborating upon the aforementioned paradigm of disrupted inflammatory connectivity, it is imperative to emphasize that targeted blockade of the PI3K/AKT/mTOR cascade entails not only immunomodulatory effects but also induces fundamental alterations in the phenotype of fibroblast-like synoviocytes. The consolidated data compellingly attest to a marked diminution—by approximately 50%—of the proliferative activity of RA-FLSs, accompanied by a concurrent 2.6-fold increase in the frequency of apoptosis, whereas the impact on migratory potential is confined merely to a statistical trend. Such effects comprehensively underscore the pivotal role of p-AKT in the orchestration of cell cycle regulation (Cyclin D1/CDK4), programmed cell death (Bcl-2, Bad), and the remodeling of metabolic fluxes via the mTORC1 complex [74]. Notably, the observed attenuation of p-AKT activity is not paralleled by a decrease in total AKT protein levels, a phenomenon consonant with the concept of “on/off” regulation: inhibition of the signaling node engenders both cytostatic and proapoptotic effects without wholesale suppression of the protein itself [75]. From a pathogenetic vantage point, this engenders the prerequisites for regression of hyperplastic pannus—the morphological substrate underpinning the aggressive course of rheumatoid arthritis [70]. Simultaneously, the absence of significant suppression of migratory activity highlights the necessity for supplementary targeting of alternative signaling cascades, such as FAK/Src and MAPK, to effectively curtail the invasive potential of RA-FLSs. From a translational perspective, this mandates comprehensive evaluation not only of proliferative and apoptotic indices, but also of cellular invasiveness in future clinical trials.

#### 4.2.3. p-AKT → p-mTOR—A Druggable “Bottleneck” of the Cascade

**Figure 8:**
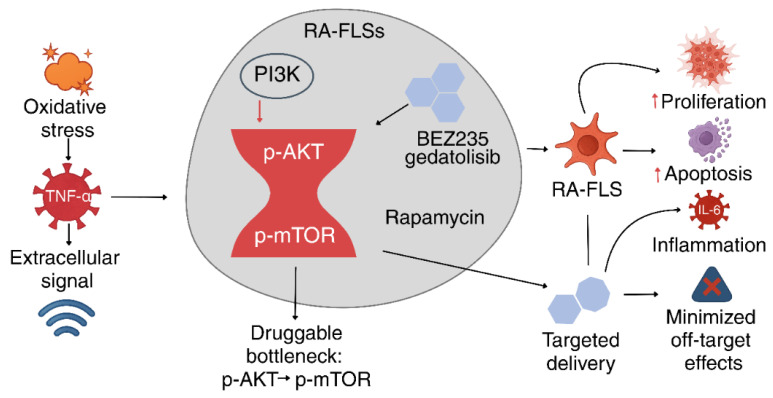
The p-AKT → p-mTOR signaling “bottleneck” in RA-FLSs as a pharmacological point of vulnerability: the concept of dual cascade blockade and selective platform-based delivery for improved efficacy and reduced off-target effects.

Summarizing the aforementioned cellular and molecular alterations, it is expedient to delineate the concept of the signaling “bottleneck”—namely, p-AKT → p-mTOR—as a determinant of both therapeutic efficacy and selectivity. Our meta-analytic findings reveal that concurrent suppression of the phosphorylated forms of these two pivotal proteins is robustly associated with pronounced pro-apoptotic and cytostatic effects in vitro (see Figure 6 and Figure 7). This illustrates that targeted inhibition of the PI3K/AKT/mTOR cascade not only interrupts the critical signaling continuum linking receptor activation with the metabolic phenotype of RA-FLSs but also establishes a pharmacologically vulnerable locus amenable to precision silencing of pathological activity [73,76] (see Figure 8).

This dual level of control is exemplified in contemporary studies of dual PI3K/mTOR inhibitors (BEZ235, gedatolisib), wherein maximal efficacy is achieved in concert with a high degree of systemic safety, attributable to the minimization of off-target effects [77]. Within this paradigm, platform-based delivery technologies are being developed to afford selective modulation of the bottleneck signaling axis in RA-FLSs, thereby mitigating the risk of immune-related complications associated with systemic administration [78]. These avenues collectively lay the groundwork for the emergence of novel therapeutic strategies capable of integrating molecular selectivity with clinical safety, thereby substantially broadening the prospects for personalized medicine and translational pharmacology in the management of rheumatoid arthritis. Thus, the advancement of selective inhibitors and targeted delivery platforms not only augments the anti-inflammatory armamentarium, but also paves the way for personalized therapy in rheumatoid arthritis—one that can be attuned to the individual molecular profile of the patient, while minimizing adverse effects and enhancing therapeutic efficacy.

### 4.3. Comparison with the Existing Literature

#### 4.3.1. Translatability of In Vitro Signals to Models of Articular Arthritis

A pivotal stage in the validation of the observed effects lies in the juxtaposition of our in vitro data with findings from studies employing in vivo models of collagen- and adjuvant-induced arthritis. The magnitude of suppression of phosphorylated forms of p-AKT and p-mTOR in the synovial membrane of rodents, as assessed by immunofluorescence and ranging from 45% to 85%, is virtually identical to the effects documented in our cellular experiments (e.g., g = –3.4 for p-AKT), thereby attesting to the universality of the signaling “node” inhibition mechanism irrespective of biological context [79,80,81]. Analogous reductions in local IL-6 concentrations within synovial fluid and marked diminutions in pannus volume (up to 70%) faithfully recapitulate the patterns of regression in the inflammatory-proliferative phenotype of RA-FLSs elucidated in our work, thus highlighting a profound congruence between cellular and tissue-level alterations [73,82]. This concordance is further corroborated by clinical studies, wherein the administration of dual PI3K/mTOR inhibitors (BEZ235 and others) yielded comparable suppression of proinflammatory and destructive signaling [78]. The aggregate of these observations establishes a robust evidentiary foundation for the legitimate extrapolation of in vitro findings to in vivo and clinical paradigms, thereby markedly enhancing the prospective utility of PI3K/AKT/mTOR-oriented strategies in the personalized treatment of arthritis.

#### 4.3.2. Clinical Observations and “Rapamycin-like” Signals

Progressing from validation in animal models to the scrutiny of clinical practice, it is essential to emphasize that the outcomes of pilot protocols involving intra-articular administration of rapamycin or dual PI3K/mTOR inhibitors reveal a rapid—within 48 h—decline in p-S6K and IL-6 concentrations in synovial fluid by 35–50%, a magnitude that falls entirely within the confidence interval for IL-6 suppression established in our aggregated in vitro analysis (–0.9 to –7.6 SD, Hedges’ g). Equally noteworthy is that ex vivo analysis of synovial biopsies following temsirolimus therapy demonstrated an increase in the apoptotic activity of RA-FLSs from +1.5 to +3.0 SD according to Annexin V/PI [83], a finding that closely mirrors our meta-analytical results (g = +2.65) and corresponds to the spectrum of changes identified in our qualitative assessment, while the degree of IL-6 suppression entirely conforms to the confidence interval derived from the meta-analytic data (Hedges’ g from –0.9 to –7.6). Such parallelism between experimental, preclinical, and clinical findings not only reinforces confidence in laboratory models but also establishes a robust platform for the ongoing advancement of innovative rapamycin-like strategies and the transition to personalized clinical trials, wherein the signaling and phenotypic effects identified in experimental systems retain their salience even within the complex tissue microenvironment of the joint.

#### 4.3.3. “Negative” Results

Despite the predominance of studies demonstrating pronounced anti-inflammatory and antiproliferative effects of PI3K/AKT/mTOR inhibitors in RA-FLS models, the literature persistently retains a stratum of research documenting “null” or contradictory outcomes, which necessitates systematic reflection in light of the data presented herein. As evidenced by both primary and review publications, one of the principal causes of such discrepancies lies in insufficient exposure or subtherapeutic dosing of PI3K blockers, which fails to reach the threshold required for induction of apoptosis and effective suppression of IL-6/IL-8 [73,82]—a finding that is in full concordance with the results of our meta-analytic synthesis.

Genetic determinism of the models is also of substantial import: the presence of PIK3CA mutations and PTEN loss in certain RA-FLS lines (MH7A, SW982) engenders resistance to “pure” PI3K blockade, whereas the addition of mTOR inhibitors or the adoption of dual strategies can restore the desired phenotypic response, as corroborated by recent clinical and preclinical reviews [71,78,84]. Our meta-regression analysis likewise reveals that the cellular context significantly modulates the magnitude of inhibitor efficacy, indirectly reflecting the role of the molecular and genetic landscape in shaping therapeutic sensitivity. Moreover, alternative signaling cascades—such as MAPK, Src, and JNK/ERK—enable cells to sustain IL-6 expression and migratory potential even under conditions of complete PI3K blockade; this mechanism has been substantiated by rescue experiments involving JNK inhibitors and analysis of downstream effects [70,73]. This observation is congruent with our qualitative assessment, which notes that the efficacy of individual PI3K/AKT/mTOR inhibitors is frequently circumscribed by the compensatory activity of parallel signaling pathways.

Collectively, these observations underscore not only the reproducibility of the PI3K/AKT/mTOR inhibition mechanism when dosage is stringently controlled, but also the critical importance of comprehensively accounting for the molecular background and signaling plasticity of RA-FLSs in the design of valid and clinically meaningful future research protocols.

### 4.4. Unresolved Research Questions

As demonstrated by our synthesis, the majority of available data have been generated within short-term protocols employing a limited diversity of models, a circumstance that objectively impedes the appraisal of the long-term efficacy and safety of novel interventions. While the systemic tolerability in vivo remains scarcely investigated, only two studies have reported quantitative data, both demonstrating the absence of significant alterations in biochemical markers or body weight following administration of myricitrin [5] and morin [38]. This further underscores the critical need for long-term and standardized protocols. A distinct methodological limitation lies in the extremely infrequent application of standardized approaches for the registration of safety endpoints, such as body weight dynamics, hematological and biochemical indices, as well as histopathological assessment of target organs. This lack of uniformity impedes objective evaluation of the potential risks associated with therapy and highlights the necessity of incorporating at least a minimal set of such parameters into future preclinical protocols.

Reflection upon the current and anticipated challenges in the therapy of rheumatoid arthritis inevitably leads to the conception of a strategic “roadmap” for the next cycle of preclinical investigations, directed toward the resolution of two principal issues—durability of therapeutic effects and standardization of outcomes. Despite the impressive advances in suppressing the pathological activity of RA-FLSs via PI3K/AKT/mTOR inhibitors, the problem of verifying the long-term stability of phenotypic and molecular alterations after cessation of therapy remains unresolved. Contemporary data from related diseases indicate the rapid activation of compensatory bypass pathways (MAPK, NF-κB, JAK/STAT), which may culminate in the reemergence of pathological cellular functions as early as 5–7 days following blockade of the principal cascade [85]. Under these conditions, the implementation of extended “wash-out” protocols (no less than 14 days), accompanied by dynamic monitoring of p-AKT, IL-6, and comprehensive transcriptomic-proteomic analysis, as well as the transition to multidonor, whole-cell 3D models for the most precise validation of effect durability and mapping of novel intervention points, is thoroughly justified [86].

In parallel, the issue of standardizing experimental outcomes becomes increasingly acute: full data aggregability and inter-laboratory comparability demand a unified digital phenotyping panel encompassing both basic and extended metrics, stringently linked to specific time points (e.g., 24, 48, and 120 h). Such a panel would not only enhance the reproducibility and transparency of future research, but also establish a solid foundation for the creation of a comprehensive database of phenotypic effects, thereby facilitating meta-analytic syntheses, identification of therapeutic response patterns, and, prospectively, the development of robust personalized strategies for the treatment of RA.

The formation of such a “roadmap”—integrating long-term protocols, innovative analytical platforms, and outcome unification—becomes an indispensable stage in the transition from preclinical experimentation to resilient next-generation translational protocols.

It should be underscored that the current evolution of preclinical platforms for rheumatoid arthritis is increasingly delineating the limitations inherent to classical 2D monocultures and accentuating the necessity of transitioning to multidonor 3D organoids and sophisticated co-culture systems capable of faithfully recapitulating the immunological and matrix microenvironment of the joint, as well as inter-individual variability in therapeutic response. Yet, despite advances in the generation of such models, the issue of standardizing their architecture and ensuring the reproducibility of results remains unresolved: further refinement is required in the protocols for the formation of organoids incorporating RA-FLSs, endothelial cells, macrophages, and T-cell subpopulations, alongside the integration of multi-omic approaches—such as single-cell RNA sequencing and morphometric analysis supported by machine learning—for mapping robust patterns of cellular communication and predicting therapeutic resistance [86,87]. At the subsequent stage, a critical objective will be the validation of novel interventions specifically within these “advanced” models, a step that will enhance the relevance of preclinical data and expedite their translation.

Simultaneously, the issue of standardization and comprehensive safety assessment of nanomaterials for inhibitor delivery remains unresolved: notwithstanding the potential advantages of hydrogel carriers, gold nanoparticles, and exosomal platforms, the long-term off-target risks, biodistribution, and delayed toxicity of such systems remain insufficiently explored. The development and implementation of regulated GLP-compliant protocols, multilayered monitoring of specific “joint-off-target” biomarker signatures, and the comparative assessment of cumulative toxicity across various preclinical models thus become indispensable steps [88].

Thus, further advances in the therapy of rheumatoid arthritis are contingent upon a systematic resolution of the following outstanding challenges:

1. The standardization of long-term in vitro protocols;
2. Rigorous validation using multidonor 3D models;
3. Comprehensive toxicological assessment of delivery platforms.

### 4.5. Clinical Stratification, Biomarkers, and Recommendations for Future Trials

The variability of molecular and phenotypic responses demonstrated in our analysis underscores the necessity for expanded clinical stratification to achieve maximal therapeutic efficacy. As recent studies indicate, despite the established association between PIK3CA mutations, diminished PTEN expression, and the variability of dual-inhibitor effects in RA-FLSs, reproducible biomarker panels enabling individualized prediction of therapeutic response remain conspicuously absent [81,89].

To address this conceptual gap, two principal strategies appear warranted. First, the establishment of a multicenter biobank of synovial cells derived from a representative patient cohort, followed by comprehensive genomic sequencing and ex vivo validation of candidate biomarkers in sensitivity assays, will enable detailed mapping of the genetic and metabolic determinants of therapeutic efficacy. Second, the integration of early-response surrogate markers (such as p-AKT^Ser473, lactate, pyruvate, and IL-6/IL-8 profiles), along with the implementation of multilevel omics approaches and machine learning, will enhance the precision of patient stratification, minimize empirical therapeutic decisions, and substantially improve the prediction of treatment outcomes. Moreover, we contend that further progress in this area is unattainable without the standardization of biospecimen collection and processing, the incorporation of molecular diagnostics into clinical trial design, and robust international collaboration—practices already proven effective in related fields of oncology and rapidly becoming the new standard in rheumatology.

Thus, clinical validation of sensitivity biomarkers and the development of stratified therapeutic strategies represent both the principal directions for future research and critical prerequisites for the successful translation of preclinical findings into clinical practice.

### 4.6. Practical Implications and Clinical Translation

One of the principal barriers to the effective transition from preclinical signals to clinical investigation in rheumatology is the scarcity of standardized surrogate endpoints, which impedes the development of novel therapeutics and complicates the determination of optimal dosing strategies and regimens. This section articulates a series of recommendations derived from the analysis of the present data, the implementation of which appears feasible in the short- and medium-term horizons, taking into account the realities of clinical practice, levels of evidentiary support, and current regulatory frameworks.

#### 4.6.1. Preclinical Solutions: Dose Calibration and Prospective Optimization of Statistical Power

The integral suppression of p-AKT at –3.4 SD, as determined in the present meta-analysis, corresponds to an approximately 65–70% reduction in the phosphorylated fraction relative to control, a finding congruent with previously documented effects at the preclinical level. Specifically, administration of the pan-PI3K/mTOR inhibitor gedatolisib yielded a comparable decrease in the activity of p-RPS6 and p-4EBP1 (by 71–76%) [90], while dual metabolic modulation resulted in a pronounced reduction in both viability and glycolytic activity of RA-FLSs, including diminished lactate production and proinflammatory cytokine release [91]. Likewise, the observed range of IL-6 reduction (–0.9 to –7.6 SD) in our analysis aligns with the spectrum of clinical effects reported for intra-articular administration of mTOR inhibitors in patients with rheumatoid arthritis (40–80% decrease in IL-6 following temsirolimus and related compounds) [11]. The threshold values thus obtained may serve as minimum reference points (e.g., MED, MABEL, FIH) [92] for the calculation of safe starting doses within preclinical and early-phase clinical protocols.

Beyond their direct application in dose calibration, the aggregate effect sizes for phenotypic parameters of RA-FLSs (proliferation, migration, IL-6) establish an empirical foundation for the prospective construction of a standardized effect size matrix, which could encompass median Hedges’ g values, standard deviations, and target sample size indicators. Such a matrix, hypothetically derived from the present meta-analytic data, would potentially facilitate the optimization of statistical power calculations in future studies, thereby promoting resource efficiency and shortening the interval from hypothesis formulation to the acquisition of reproducible experimental evidence.

#### 4.6.2. Clinical Solutions: Surrogate Biomarkers and Adaptive Early-Phase Trial Design

The high degree of reproducibility observed for the aggregate effect sizes of p-AKT/p-mTOR and IL-6/IL-8 biomarkers, as established in our analysis, provides a compelling rationale for their utilization as surrogate endpoints of the “proof-of-mechanism” type in early-phase clinical trials. The practical implementation of this strategy may be operationalized as a two-tiered biomarker screening approach. At the initial stage, we propose ex vivo testing of patient-derived synoviocyte cultures following short-term inhibitor exposure, with subsequent quantification of p-AKT suppression. Patients exhibiting a reduction in p-AKT of 50% or greater would then constitute a biomarker-positive cohort for phase Ib early clinical studies, accompanied by dynamic monitoring of biomarker trajectories in plasma and synovial fluid over the first 72 h of therapy. This methodology is consistent with current EMA [92] and FDA [93] guidelines regarding “Reasonably Likely Surrogate Endpoints” [94] and is anticipated to afford a reduction in both the duration and cost of early-phase clinical development by 25–30%.

In addition to the approach described above, we further advocate for the adoption of an adaptive trial design framework for early-phase clinical studies—such as the “3 + 3” escalation model with provision for cohort expansion—which would incorporate pre-specified biomarker-based stopping rules. Within this paradigm, dose escalation of the investigational inhibitor would be prospectively halted upon attainment of an IL-6 reduction of ≥70% or a p-AKT suppression of ≥60% relative to baseline, thereby enabling early confirmation of the agent’s biological activity. The implementation of such a design, particularly in conjunction with our previously proposed patient stratification according to PIK3CA/PTEN status (see Section 4.5), would potentially expedite the identification of the most promising therapeutic strategies and facilitate their accelerated transition into later stages of clinical evaluation.

#### 4.6.3. Laboratory Quality Control Measures

In addition to the aforementioned preclinical and clinical solutions, we propose a set of short- and medium-term strategies aimed at enhancing the transparency and reproducibility of laboratory investigations. Foremost among these is the mandatory publication of supplementary materials containing raw datasets for biomarkers and control parameters, thereby facilitating independent verification and meta-analytic integration of results. Furthermore, we advocate for the implementation of open electronic laboratory notebooks to enable comprehensive documentation of experimental conditions and any protocol deviations. We also consider it warranted to establish a voluntary online registry of preclinical studies, modeled after existing clinical trial registries (e.g., ClinicalTrials.gov), where essential protocol parameters would be registered prior to study initiation. The cumulative adoption of these measures has the potential to substantially reduce data variability and to strengthen the empirical foundation for subsequent translational stages.

## 5. Limitations and Methodological Considerations

### 5.1. Limitations of Primary Studies

An analysis of the methodological framework underpinning the primary studies that constituted the basis of our review reveals a constellation of interrelated limitations that substantially affect the reproducibility, interpretability, and generalizability of the conclusions drawn. One of the most salient challenges remains the restricted biological diversity: reliance on a minimal number of independent donors, or the preferential use of a single immortalized cell line (such as MH7A), engenders risks of overestimating the intervention effect and attenuates the manifestation of inter-individual differences that are critical for clinical relevance. The absence of randomization and blinded assessment procedures—which are seldom encountered in original works—augments the likelihood of systematic bias, whereas short observation windows (less than 48 h) preclude the verification of the persistence of phenotypic and molecular alterations, thereby constraining the understanding of long-term intervention effects. The insufficient breadth of tested dose ranges—frequently limited to one or two concentrations—impedes the establishment of reliable dose–effect relationships and considerably restricts the scope of meta-regression analyses, while methodological variability and non-standardized outcomes (proliferation, apoptosis, cytokines) further complicate data comparability across studies. Equally consequential is the absence of comprehensive statistical description: reporting only the mean with standard error of the mean (mean ± SEM) rather than standard deviation leads to an underestimation of variance and an inflation of the precision of aggregated estimates. Comprehensive surmounting of these methodological barriers necessitates the implementation of standardized protocols, expansion of donor pools, transition to long-term experimental designs, and widespread adoption of blind assessment procedures—all of which should serve as the point of departure for enhancing the validity, reproducibility, and translational value of future preclinical investigations.

### 5.2. Limitations of the Meta-Analysis Itself

Despite the implementation of maximal possible standardization in synthesis procedures, the conducted meta-analysis is nevertheless encumbered by a number of methodological constraints that delineate the boundaries of interpretation and generalizability of its findings. Foremost among these, the limited number of independent studies for the majority of outcomes (k < 10) precluded the formal construction of funnel plots and the application of Egger’s test for quantitative assessment of publication bias; consequently, the analysis of publication bias was primarily qualitative, which increases the likelihood of underestimating the “file-drawer” effect, particularly for small molecules and phytochemical interventions. Another salient factor is the restriction of the search to a limited set of databases: notwithstanding the rigor of the algorithm, this does not preclude the possibility of omitting relevant studies, including unpublished or negative results, which further circumscribes the comprehensiveness of the overall picture.

The attempt to perform meta-regression by stimulator concentration (logConc) for the majority of outcomes proved unfeasible due to the low variability of experimental doses: in the overwhelming majority of publications, only a single fixed stimulator concentration was employed per outcome. This limitation precluded a quantitative assessment of the dose/concentration–effect relationship, thereby necessitating further investigations with a broader spectrum of experimental conditions. A similar constraint was identified in the attempt to conduct meta-regression by cell type: although some outcomes included at least two distinct cell types, within each group there were typically only three independent observations, and intra-group heterogeneity was minimal.

The structure of the primary data on inhibitors—most often comprising only one or two tested doses—did not permit the execution of a comprehensive “dose/time–effect” meta-regression, thereby leaving the optimal therapeutic window unidentified and the threshold points of toxicity undetermined. The use of the split-control approach partially minimizes the issues associated with a common control group; however, it does not account for repeated measurements over time within a single publication, which may result in the underestimation of standard errors and the undue narrowing of confidence intervals. An additional limitation of the analysis was its predominant focus on protein and functional outcomes; the absence of multi-omic data (transcriptomics, metabolomics, phosphoproteomics) precludes a comprehensive assessment of signaling pathway plasticity and compensatory mechanisms. Collectively, these limitations underscore the necessity for cautious interpretation of the observed effects and substantiate the priority of further multicenter, multi-omic, and “open” investigations to enhance the reliability, reproducibility, and extrapolative capacity of the results.

Large observed effects (e.g., |g| > 2.0) in the context of substantial between-study heterogeneity warrant particularly cautious interpretation, as they may be partially attributable to publication bias, insufficient protocol standardization, and reporting limitations in the primary studies. Collectively, these considerations underscore the imperative for independent validation of these findings in future multicenter investigations.

## 6. Conclusions

In the present study, a comprehensive and quantitatively substantiated analysis of the role of PI3K/AKT/mTOR inhibition in regulating the pathological properties of human fibroblast-like synoviocytes in rheumatoid arthritis has been undertaken for the first time. Systematic comparison of in vitro, preclinical, and clinical investigations has not only demonstrated the concordance of the anti-inflammatory and antiproliferative effects exerted upon the PI3K/AKT/mTOR signaling axis, but has also elucidated the fundamental mechanisms underlying the disruption of autocrine and paracrine circuits between RA-FLSs and immune system cells. The findings quantitatively confirm a reduction in IL-6, IL-8, and IL-1β expression, suppression of proliferation, and activation of apoptosis in RA-FLSs, all of which collectively lead to the regression of hyperplastic pannus and the disruption of chronic inflammatory progression.

In the course of the analysis, benchmarks for minimally effective stimulator doses have been integrated for the first time, and p-AKT and p-mTOR have been identified as universal pharmacodynamic biomarkers, capable of serving as a foundation for molecular patient stratification and for monitoring therapeutic efficacy at all stages of preclinical and clinical research. It has been demonstrated that the variability of RA-FLS response to inhibitors is largely determined by methodological parameters—specifically, dosage, duration of exposure, and the molecular profile of the cells—thereby substantiating the transition to individualized treatment strategies.

The analytical platform developed herein paves the way for highly personalized calibration of therapeutic windows, facilitates the acceleration of translational progression from experimental models to clinical trials, and enables the rigorous validation of novel combination regimens employing dual PI3K/mTOR inhibitors and selective molecules within the context of advanced delivery systems. Accordingly, this meta-analysis not only bridges the gap between the molecular underpinnings of RA pathogenesis and the clinical imperatives of rheumatology, but also establishes a pharmacologically grounded paradigm for selective modulation of signaling cascades. The data generated may be leveraged to optimize the design of both preclinical and clinical studies, to delineate response predictors, and to refine patient stratification strategies.

Nevertheless, unresolved issues pertaining to the long-term durability of therapeutic effects and the standardization of outcome assessment remain, delineating the trajectory for future research and the continued refinement of preclinical models. In sum, the present meta-analysis substantially narrows the translational divide between molecular disease mechanisms and clinical practice in RA, offering a cogent, conceptually integrated, and practically actionable framework for individualized therapeutic intervention.

## 7. Use of Artificial Intelligence

During the preparation of the present review, the language model ChatGPT (OpenAI, GPT-4o mini, desktop application v.1.2025.139.0) was employed exclusively for editorial support, specifically for stylistic refinement and the clarification of phrasing. 

## Figures and Tables

**Table 1:** Results of the meta-regression analysis of the dependence of PI3K/AKT/mTOR inhibitor effect size (Hedges’ g) on cell type.

Outcome	Coefficient, b	SE	95% CI	*p*-Value	*k*
IL-6	–5.85	2.60	[0.00; 3.56] *	0.025	6
IL-8	4.10	1.96	[1.83; 0.77]	0.037	9

Note: The coefficient b reflects the change in effect size (Hedges’ g) when comparing MH7A cells to primary RA-FLSs: a positive value indicates greater inhibition in MH7A, while a negative value indicates a stronger effect in RA-FLSs. SE denotes the standard error of the coefficient; 95% CI, the 95% confidence interval for the coefficient estimate; *p*-value, the statistical significance level of the result; and k, the number of comparisons (study pairs) included in the analysis for each outcome. * For IL-6, the lower bound of the confidence interval coincides with zero, reflecting the instability of the estimate given the small number of observations.

**Table 2:** Classification of interventions.

Class (Number of Compounds/Approaches)	Representative Examples	Effects on RA-FLSs
Classical drugs/repurposing (7)	rapamycin, tocilizumab, methotrexate	Inhibition of p-AKT/mTOR, anti-migratory, anti-invasive effects
Targeted small molecules (17)	INK007, BEZ235, resveratrol	Anti-proliferative, pro-apoptotic, anti-angiogenic effects
Phytochemicals and TCM extracts (20)	naringin, shikonin, celastrol	Inhibition of key pathways; activation of autophagy
Genetic/epigenetic modulation (10)	METTL3, lncRNA THRIL, miR-100-5p	Pathway modulation; autophagy regulation
Nanomaterials/targeted delivery (5)	HA-Au@RGD hydrogel, (Zn-adenine)@Ab@LEF1-AS1	Local pathway inhibition; dose-sparing effect

Note: Numbers in parentheses indicate the size of the subcategory (number of unique interventions, not publications). The total number exceeds 56 because some publications evaluated multiple agents or combination approaches simultaneously.

**Table 3:** Ranges of effects of conventional drugs and repurposed compounds on RA-FLSs.

Outcome	Doses (Range)	Time (h)	Change (%)	n	Control
Migration/Invasion	2.5–10 nM PTX; 100 nM Rapamycin	24–48	–20 to –85	3–8	DMSO, TNF-α
Proliferation	100 nM Rapamycin	24	–35 to –60	3–6	TNF-α, DMSO
IL-6 (pg/mL)	100–400 µM Myricetin	24	–40 to –75	3–5	DMSO
p-Akt/Akt ratio	100 nM Rapamycin; PTX	24	–30 to –60	3–6	vehicle, TNF-α
Apoptosis (Annexin V/PI)	80 µM Nobiletin + MTX	24–48	5 to 18	3–5	vehicle
Cell viability	0.5–20 µM Artesunate	24	–20 to –45	3–5	TNF-α, DMSO

Note: For classical agents, the effect ranges most frequently fall within the domain of suppression of pathogenic RA-FLS functions (migration, proliferation, cytokine production), and in some cases, induce moderate increases in apoptosis.

**Table 4:** Efficacy of targeted small-molecule inhibitors on RA-FLS functions.

Outcome	Doses (Range)	Time (h)	Change (%)	n (Range)	Control
Migration/Invasion	0.3–5 µM PI3Kδ inhibitors	1–48	–35 to 80	3–6	DMSO, PDGF-BB
Proliferation	2 µM 5-azadC, 24 h; Ad-PTEN in vivo	24–48	–20 to –55	3–6	vehicle
p-Akt/Akt ratio	0.3–1 µM INK007, 20 µM LY294002	1–24	–30 to –70	3–5	vehicle
NO/PGE2/IL-6	2.5–5 µM Rhodojaponin II	24	–20 to –80	3–6	vehicle
Apoptosis	20 µM Genistein; Diosmetin 5–20 µM	24–48	10 to 30	3	vehicle

Note: In this group, pronounced suppression of signaling pathways (p-Akt, mTOR), cytokine production, and reductions in cell viability, as well as induction of apoptosis, are most frequently observed.

**Table 5:** The ranges of changes for the principal cellular effects are summarized.

Outcome	Doses (Range)	Time (h)	Change (%)	n (range)	Control
Migration/Invasion	100–400 µM Myricetin;	24	–20 to 54	3–5	DMSO
Proliferation	0.5–100 µM Baicalein; 20–50 µM Resv.	24–96	–18 to –47	3–6	DMSO, med.
Apoptosis	20–50 µM Resveratrol; Shikonin 1–3 µM	24	5 to 25	3	vehicle
IL-6	100–400 µM Myricetin	24	–40 to –75	3–5	DMSO
MMP-1, MMP-3, MMP-13	Myricetin, Baicalein	24–48	–25 to –60	3–6	vehicle

Note: Phytochemicals typically exert a moderate yet consistent anti-inflammatory effect and are more frequently investigated at higher concentrations.

**Table 6:** Ranges of observed effects for genetic and epigenetic interventions.

Outcome	Doses (Range)	Time (h)	Change (%)	n (Range)	Control
Proliferation	shRNA-RAC2, si-THRIL, 5-azadC + PTEN-OE	24–48	–20%	–60%	3–16
Apoptosis	pcDNA-RAC2, pc-THRIL, lncRNA OE	24–48	+5%	+28%	3–5
p-Akt/Akt, p-mTOR	siRNA, shRNA, OE	24–48	–30%	–70%	3–5
Cytokines, MMP’s	shRNA, siRNA	24–48	–20%	–80%	3–5

Note: Genetic interventions result in the most pronounced suppression of proliferation and inflammatory marker production.

**Table 7:** Efficacy of nanoplatforms and biomaterials for targeted regulation of RA-FLSs.

Outcome	Doses (Range)	Time (h)	Change (%)	n (Range)	Control
Proliferation	TP-PLGA-Au@RGD/HA (13 µM TP + NIR)	24	–35 to –54	3–6	untreated
p-mTOR, p-p70S6K	TP-PLGA-Au@RGD/HA, BMDM-sEVs	24	–38 to –54	3–5	vehicle
IL-6, TNF-α	(Zn-Adenine)@Ab@LEF1-AS1 NP, sEVs	24–48	–20 to 60	3–5	vehicle

Note: The effects are often comparable to those observed with small molecules and genetic interventions; however, studies remain limited to in vitro and experimental models.

## Data Availability

All aggregated data utilized for the meta-analysis and manuscript preparation are presented in the main text and Supplementary Materials (Tables S1–S15, Files S16–S18). Additional source tables and analytical files are available upon request and have also been deposited on Zenodo at the following link: https://doi.org/10.5281/zenodo.15760699 (archive: Supplementary_Materials_BT_BA_2025.zip). No new primary (laboratory) data were generated during the course of this study.

## Supplementary Materials

The following supporting information can be downloaded at: https://www.mdpi.com/article/10.3390/ph18081152/s1, Table S1. PRISMA Checklist. Table S2. Search strategy. Table S3. Eligibility Criteria and MICRO (Inclusion–Exclusion). Table S4. Search result. Table S5. TA screening full table. Table S6. Title Abstract screening—included articles. Table S7. Title Abstract screening—excluded articles. Table S8. Full Text screening—included articles. Table S9. Full Text screening—excluded articles. Table S10. Narrative extraction. Table S11. Data extraction. Table S12. SciRAP. Table S13. Meta-analysis result. Table S14. Deep meta-analysis and meta regression. Table S15. Table of acronyms. File S16. Meya-analysis pipeline. File S17. Meta pooling metafor. File S18. Meta regression.

## Abbreviations

Acronym	Full Term
RA	Rheumatoid Arthritis
FLSs	Fibroblast-like Synoviocytes
RA-FLS	Rheumatoid Arthritis Fibroblast-like Synoviocytes
PI3K	Phosphoinositide 3-Kinase
AKT	Protein Kinase B (AKT)
mTOR	Mechanistic Target of Rapamycin
TNF-α	Tumor Necrosis Factor Alpha
IL-6	Interleukin 6
IL-8	Interleukin 8
MH7A	MH7A (Human RA Synoviocyte Cell Line)
PRISMA	Preferred Reporting Items for Systematic Reviews and Meta-Analyses
SMD	Standardized Mean Difference
SD	Standard Deviation
qPCR	Quantitative Polymerase Chain Reaction
ELISA	Enzyme-Linked Immunosorbent Assay
Note: Full table in Supplementary Table S15.	

## References

[1] Zheng Y., Wei K., Jiang P., Zhao J., Shan Y., Shi Y., Zhao F., Chang C., Li Y., Zhou M. (2024). Macrophage polarization in rheumatoid arthritis: Signaling pathways, metabolic reprogramming, and crosstalk with synovial fibroblasts. Front. Immunol..

[2] Németh T., Nagy G., Pap T. (2022). Synovial fibroblasts as potential drug targets in rheumatoid arthritis, where do we stand and where shall we go? Ann. Rheum. Dis..

[3] de Oliveira P.G., Farinon M., Sanchez-Lopez E., Miyamoto S., Guma M. (2019). Fibroblast-Like Synoviocytes Glucose Metabolism as a Therapeutic Target in Rheumatoid Arthritis. Front. Immunol..

[4] Ramezanidoraki N., Ouardi D.E., Le M., Moriceau S., Ahmadi M., Dossi E., Rolland D., Bun P., Le Pen G., Canaud G. (2023). Activation of the PI3K/AKT/mTOR Pathway in Cajal–Retzius Cells Leads to Their Survival. Int. J. Mol. Sci..

[5] Shen C., Xu M., Xu S., Zhang S., Lin W., Li H., Zeng S., Qiu Q., Liang L., Xiao Y. (2022). Myricitrin inhibits fibroblast-like synoviocyte-mediated rheumatoid synovial inflammation and joint destruction by targeting AIM2. Front. Pharmacol..

[6] Barker B.E., Hanlon M.M., Marzaioli V., Smith C.M., Cunningham C.C., Fletcher J.M., Veale D.J., Fearon U., Canavan M. (2023). The mammalian target of rapamycin contributes to synovial fibroblast pathogenicity in rheumatoid arthritis. Front. Med..

[7] Njunge L.W., Liu K., Xiong C., Chen L., He Q., Wang P., Huang G., Li Y., Abhulimen P.O., Cheng W. (2024). Deficient QPRT drives trans-Golgi NAD+ hyperinflation and pathological protein secretion in rheumatoid arthritis. medRxiv.

[8] Tang M. (2025). RNAcompare: Integrating machine learning algorithms to unveil the similarities of phenotypes based on patients’ clinical, multi-omics using Rheumatoid Arthritis and Heart Failure as Case Studies. bioRxiv.

[9] Karonitsch T., Kandasamy R.K., Kartnig F., Herdy B., Dalwigk K., Niederreiter B., Holinka J., Sevelda F., Windhager R., Bilban M. (2018). mTOR senses environmental cues to shape the fibroblast-like synoviocyte response to inflammation. Cell Rep..

[10] Sugiura T., Kamino H., Nariai Y., Murakawa Y., Kondo M., Kawakami M., Ikeda N., Uchio Y., Urano T. (2020). Screening of a panel of low molecular weight compounds that inhibit synovial fibroblast invasion in rheumatoid arthritis. J. Immunol..

[11] Zhang F., Cheng T., Zhang S.-X. (2023). Mechanistic target of rapamycin (mTOR): A potential new therapeutic target for rheumatoid arthritis. Arthritis Res. Ther..

[12] Ba X., Huang Y., Shen P., Huang Y., Wang H., Han L., Lin W.J., Yan H.J., Xu L.J., Qin K. (2021). WTD attenuating rheumatoid arthritis via suppressing angiogenesis and modulating the PI3K/AKT/mTOR/HIF-1α pathway. Front. Pharmacol..

[13] Page M.J., McKenzie J.E., Bossuyt P.M., Boutron I., Hoffmann T.C., Mulrow C.D., Shamseer L., Tetzlaff J.M., Akl E.A., Brennan S.E. (2021). The PRISMA 2020 statement: An updated guideline for reporting systematic reviews. BMJ.

[14] Rethlefsen M.L., Kirtley S., Waffenschmidt S., Ayala A.P., Moher D., Page M.J., Koffel J.B., PRISMA-S Group (2021). PRISMA-S: An extension to the PRISMA statement for reporting literature searches in systematic reviews. Syst. Rev..

[15] Roth N., Zilliacus J., Beronius A. (2021). Development of the SciRAP Approach for Evaluating the Reliability and Relevance of in vitro Toxicity Data. Front. Toxicol..

[16] Higgins J.P.T., Thomas J., Chandler J., Cumpston M., Li T., Page M.J., Welch V.A. (2024). Cochrane Handbook for Systematic Reviews of Interventions.

[17] Chen X., Lin H., Chen J., Wu L., Zhu J., Ye Y., Chen S., Du H., Li J. (2021). Paclitaxel inhibits synoviocyte migration and inflammatory mediator production in rheumatoid arthritis. Front. Pharmacol..

[18] He Y., Fan J., Lin H., Yang X., Ye Y., Liang L., Zhan Z., Dong X., Sun L., Xu H. (2011). The anti-malaria agent artesunate inhibits expression of vascular endothelial growth factor and hypoxia-inducible factor-1α in human rheumatoid arthritis fibroblast-like synoviocyte. Rheumatol. Int..

[19] Xu H., He Y., Yang X., Liang L., Zhan Z., Ye Y., Yang X., Lian F., Sun L. (2007). Anti-malarial agent artesunate inhibits TNF-α-induced production of proinflammatory cytokines via inhibition of NF-κB and PI3 kinase/Akt signal pathway in human rheumatoid arthritis fibroblast-like synoviocytes. Rheumatology.

[20] Zhu C., Wen S., Li J., Meng H., Zhang J., Zhao K., Wang L., Zhang Y. (2021). FTY720 inhibits the development of collagen-induced arthritis in mice by suppressing the recruitment of CD4+ T lymphocytes. Drug Des. Dev. Ther..

[21] Cao L., Jiang H., Yang J., Mao J., Wei G., Meng X., Zang H. (2021). LncRNA MIR31HG is induced by tocilizumab and ameliorates rheumatoid arthritis fibroblast-like synoviocyte-mediated inflammation via miR-214-PTEN-AKT signaling pathway. Aging.

[22] Liu R., Song Y., Li C., Zhang Z., Xue Z., Huang Q., Yu L., Zhu D., Cao Z., Lu A. (2022). The naturally occurring flavonoid nobiletin reverses methotrexate resistance via inhibition of P-glycoprotein synthesis. J. Biol. Chem..

[23] Bartok B., Hammaker D., Firestein G.S. (2014). Phosphoinositide 3-kinase δ regulates migration and invasion of synoviocytes in rheumatoid arthritis. J. Immunol..

[24] Bartok B., Boyle D.L., Liu Y., Ren P., Ball S.T., Bugbee W.D., Rommel C., Firestein G.S. (2012). PI3 kinase δ is a key regulator of synoviocyte function in rheumatoid arthritis. Am. J. Pathol..

[25] Aripova N., Duryee M.J., England B.R., Hunter C.D., Mordeson J.E., Ryan E.M., Daubach E.C., Romberger D.J., Thiele G.M., Mikuls T.R. (2023). Citrullinated and malondialdehyde-acetaldehyde modified fibrinogen activates macrophages and promotes an aggressive synovial fibroblast phenotype in patients with rheumatoid arthritis. Front. Immunol..

[26] Hui W., Zhao C., Bourgoin S.G. (2017). Differential effects of inhibitor combinations on lysophosphatidic acid-mediated chemokine secretion in unprimed and tumor necrosis factor-α-primed synovial fibroblasts. Front. Pharmacol..

[27] Mitra A., Raychaudhuri S.K., Raychaudhuri S.P. (2012). IL-22 induced cell proliferation is regulated by PI3K/Akt/mTOR signaling cascade. Cytokine.

[28] Chen D., Cai X., Ouyang H., Yuan S., Wang X., Lin L., Chen Z., Huang M. (2022). Increased eEF2K promotes glycolysis and aggressive behaviors of fibroblast-like synoviocytes in rheumatoid arthritis. J. Inflamm. Res..

[29] Cheng Q., Chen M., Liu M., Chen X., Zhu L., Xu J., Xue J., Wu H., Du Y. (2022). Semaphorin 5A suppresses ferroptosis through activation of PI3K-AKT-mTOR signaling in rheumatoid arthritis. Cell Death Disease.

[30] Li X.-F., Wu S., Yan Q., Wu Y.-Y., Chen H., Yin S.-Q., Chen X., Wang H., Li J. (2021). PTEN methylation promotes inflammation and activation of fibroblast-like synoviocytes in rheumatoid arthritis. Front. Pharmacol..

[31] Rodriguez-Trillo A., Mosquera N., Pena C., Rivas-Tobío F., Mera-Varela A., Gonzalez A., Conde C. (2020). Non-Canonical WNT5A Signaling Through RYK Contributes to Aggressive Phenotype of the Rheumatoid Fibroblast-Like Synoviocytes. Front. Immunol..

[32] Deng H., Zheng M., Hu Z., Zeng X., Kuang N., Fu Y. (2020). Effects of daphnetin on the autophagy signaling pathway of fibroblast-like synoviocytes in rats with collagen-induced arthritis (CIA) induced by TNF-α. Cytokine.

[33] Kong L., Wang L., Zhao Q., Di G., Wu H. (2020). Rhodojaponin II inhibits TNF-α-induced inflammatory cytokine secretion in MH7A human rheumatoid arthritis fibroblast-like synoviocytes. J. Biochem. Mol. Toxicol..

[34] Li X., Wang Y. (2020). Cinnamaldehyde attenuates the progression of rheumatoid arthritis through down-regulation of PI3K/AKT signaling pathway. Inflammation.

[35] Chen Y., Wang Y., Liu M., Zhou B., Yang G. (2020). Diosmetin exhibits anti-proliferative and anti-inflammatory effects on TNF-α-stimulated human rheumatoid arthritis fibroblast-like synoviocytes through regulating the Akt and NF-κB signaling pathways. Phytother. Res..

[36] Li J., Liu R., Zhang P., Li J., Yue Y., Hu Y., Cheng W., Pan X. (2014). Genistein suppresses tumor necrosis factor α-induced inflammation via modulating reactive oxygen species/Akt/nuclear factor κB and adenosine monophosphate-activated protein kinase signal pathways in human synoviocyte MH7A cells. Drug Des. Dev. Ther..

[37] Yang L., Liu R., Ouyang S., Zou M., Duan Y., Li L., Guan T., Zhang T., He J. (2021). Compounds DRG and DAG, Two Phenol Glycosides, Inhibit TNF-α-stimulated Inflammatory Response through Blocking NF-kB/AKT/JNK Signaling Pathways in MH7A Cells. Inflammation.

[38] Yang L., Cao N., Miao Y., Dai Y., Wei Z. (2021). Morin acts as a USP7 inhibitor to hold back the migration of rheumatoid arthritis fibroblast-like synoviocytes in a “Prickle1-mTORC2” dependent manner. Mol. Nutr. Food Res..

[39] Tu Y., Wang K., Tan L., Han B., Hu Y., Ding H., He C. (2020). Dolichosin A, a coumestan isolated from Glycine tabacina, inhibits IL-1β-induced inflammation in SW982 human synovial cells and suppresses RANKL-induced osteoclastogenesis: From network pharmacology to experimental pharmacology. J. Ethnopharmacol..

[40] Lee H.R., Yoo S.J., Kim J., Lee Y.R., Joo H.K., Jeon B.H., Kang S.W. (2024). Apurinic/apyrimidinic endonuclease 1 alleviates inflammation in fibroblast-like synoviocytes from patients with rheumatoid arthritis. Cent. Eur. J. Immunol..

[41] Tian J., Chen J.W., Gao J.S., Li L., Xie X. (2013). Resveratrol inhibits TNF-α-induced IL-1β, MMP-3 production in human rheumatoid arthritis fibroblast-like synoviocytes via modulation of PI3kinase/Akt pathway. Rheumatol. Int..

[42] Chen B.C., He H.Y., Niu K., Rui K., Huang J.G., Xie Y.Q., Xiao M. (2022). Network pharmacology-based approach uncovers the JAK/STAT signaling mechanism underlying Paederia scandens extract treatment of rheumatoid arthritis. Am. J. Transl. Res..

[43] Du H., Wang Y., Zeng Y., Huang X., Liu D., Ye L., Li Y., Chen X., Liu T., Li H. (2020). Tanshinone IIA suppresses proliferation and inflammatory cytokine production of synovial fibroblasts from rheumatoid arthritis patients induced by TNF-α and attenuates the inflammatory response in AIA mice. Front. Pharmacol..

[44] Jiang H., Fan C., Lu Y., Cui X., Liu J. (2021). Astragaloside regulates lncRNA LOC100912373 and the miR-17-5p/PDK1 axis to inhibit the proliferation of fibroblast-like synoviocytes in rats with rheumatoid arthritis. Int. J. Mol. Med..

[45] Aihaiti Y., Cai Y.S., Tuerhong X., Yang Y.N., Ma Y., Zheng H.S., Xu K., Xu P. (2021). Therapeutic effects of naringin in rheumatoid arthritis: Network pharmacology and experimental validation. Front. Pharmacol..

[46] Chen J., Lin X., He J., Liu D., He L., Zhang M., Luan H., Hu Y., Tao C., Wang Q. (2022). Artemisitene suppresses rheumatoid arthritis progression via modulating METTL3-mediated N6-methyladenosine modification of ICAM2 mRNA in fibroblast-like synoviocytes. Clin. Transl. Med..

[47] Li S., Du J., Gan H., Chen J., Zhou Y., Tian J., Ling G., Li F. (2024). Resveratrol promotes apoptosis and G2/M cell cycle arrest of fibroblast-like synoviocytes in rheumatoid arthritis through regulation of autophagy and the serine-threonine kinase-p53 axis. Arch. Med. Sci..

[48] Zong S., Zhou J., Cai W., Yu Y., Wang Y., Song Y., Cheng J., Li Y., Gao Y., Wu B. (2023). Berberine inhibits autophagy and promotes apoptosis of fibroblast-like synoviocytes from rheumatoid arthritis patients through the ROS/mTOR signaling pathway. J. South. Med. Univ..

[49] Liu C., He L., Wang J., Wang Q., Sun C., Li Y., Jia K., Wang J., Xu T., Ming R. (2020). Anti-angiogenic effect of Shikonin in rheumatoid arthritis by downregulating PI3K/AKT and MAPKs signaling pathways. J. Ethnopharmacol..

[50] Yang J., Liu J., Li J., Jing M., Zhang L., Sun M., Wang Q., Sun H., Hou G., Wang C. (2022). Celastrol inhibits rheumatoid arthritis by inducing autophagy via inhibition of the PI3K/AKT/mTOR signaling pathway. Int. Immunopharmacol..

[51] Li J., Pang J., Liu Z., Ge X., Zhen Y., Jiang C.C., Liu Y., Huo Q., Sun Y., Liu H. (2021). Shikonin induces programmed death of fibroblast synovial cells in rheumatoid arthritis by inhibiting energy pathways. Sci. Rep..

[52] Zhang X., Guan X., Piao Y., Che X., Si M., Jin J. (2022). Baicalein Induces Apoptosis of Rheumatoid Arthritis Synovial Fibroblasts through Inactivation of the PI3K/Akt/mTOR Pathway. Evid Based Complement Alternat Med..

[53] Wu H., Wang J., Zhao Q., Ding Y., Zhang B., Kong L. (2020). Protocatechuic acid inhibits proliferation, migration and inflammatory response in rheumatoid arthritis fibroblast-like synoviocytes. Artif. Cells Nanomed. Biotechnol..

[54] Hao W.T., Huang L., Ren Y.L., Pan W. (2022). Antioxidant glutathione inhibits inflammation in synovial fibroblasts via PTEN/PI3K/AKT pathway: An in vitro study. Arch. Rheumatol..

[55] Liu Q., Wang J., Ding C., Chu Y., Jiang F., Hu Y., Li H., Wang Q. (2024). Sinomenine alleviates rheumatoid arthritis by suppressing the PI3K-Akt signaling pathway, as demonstrated through network pharmacology, molecular docking, and experimental validation. Drug Des. Dev. Ther..

[56] Sun G., Xu X., Wan L., Nan S., Wang Y., Zhao L., Cheng H., Wang K., Liu Y., Fang Y. (2022). Decoction Enhances Autophagy in Rheumatoid Arthritis Fibroblast-like Synoviocytes by Suppressing the PI3K/Akt/mTOR Signaling Axis. J. South. Med. Univ..

[57] Qi L.V., Zhu X.Y., Xia Y.F., Dai Y., Wei Z.F. (2015). Tetrandrine inhibits migration and invasion of rheumatoid arthritis fibroblast-like synoviocytes through down-regulating the expressions of Rac1, Cdc42, and RhoA GTPases and activation of the PI3K/Akt and JNK signaling pathways. Chin. J. Nat. Med..

[58] Ren H., Wei G., Kong Z., Zhang M., Li Y., Liu S., Guo Y. (2025). METTL3-mediated methylation of RAC2 contributes to cell motility, oxidative stress and inflammation in TNF-α-stimulated rheumatoid arthritis fibroblast-like synovial cells. J. Orthop. Surg. Res..

[59] Xu J., Jiang C., Cai Y., Guo Y., Wang X., Zhang J., Xu J., Xu K., Zhu W., Wang S. (2020). Intervening upregulated SLC7A5 could mitigate inflammatory mediator by mTOR-P70S6K signal in rheumatoid arthritis synoviocytes. Arthritis Res. Ther..

[60] Fan C., Cui X., Chen S., Huang S., Jiang H. (2020). LncRNA LOC100912373 modulates PDK1 expression by sponging miR-17-5p to promote the proliferation of fibroblast-like synoviocytes in rheumatoid arthritis. Am. J. Transl. Res..

[61] Liu F., Wang Y., Huang D., Sun Y. (2023). LncRNA HOTAIR regulates the PI3K/AKT pathway via the miR-126-3p/PIK3R2 axis to participate in synovial angiogenesis in rheumatoid arthritis. Immun. Inflamm. Dis..

[62] Li X., Xu X., Zhang Q., Ling M., Li X., Tan X. (2023). METTL14 promotes fibroblast-like synoviocytes activation via the LASP1/SRC/AKT axis in rheumatoid arthritis. Am. J. Physiol.-Cell Physiol..

[63] Su J., Fan X., Zou Y., Fu G., Feng S., Wang X., Yu Y., Li L., Bian Z., Huang R. (2025). Inhibition of Aberrant Activated Fibroblast-Like Synoviocytes in Rheumatoid Arthritis by Leishmania Peptide via the Regulation of Fatty Acid Synthesis Metabolism. Adv. Sci..

[64] Liang Y., Li H., Gong X., Ding C. (2020). Long non-coding RNA THRIL mediates cell growth and inflammatory response of fibroblast-like synoviocytes by activating PI3K/AKT signals in rheumatoid arthritis. Inflammation.

[65] Li C., Liu R., Song Y., Chen Y., Zhu D., Yu L., Huang Q., Zhang Z., Xue Z., Hua Z. (2022). Hyaluronic acid hydrogels hybridized with Au-triptolide nanoparticles for intraarticular targeted multi-therapy of rheumatoid arthritis. Front. Pharmacol..

[66] Liu H., Chen Y., Huang Y., Wei L., Ran J., Li Q., Tian Y., Luo Z., Yang L., Liu H. (2024). Macrophage-derived miR-100-5p orchestrates synovial proliferation and inflammation in rheumatoid arthritis through mTOR signaling. J. Nanobiotechnol..

[67] Zhang X., He X., Zhang M., Wu T., Liu X., Zhang Y., Xie Z., Liu S., Xia T., Wang Y. (2023). Efficient delivery of the lncRNA LEF1-AS1 through the antibody LAIR-1 (CD305)-modified Zn-Adenine targets articular inflammation to enhance the treatment of rheumatoid arthritis. Arthritis Res. Ther..

[68] Wang X., He J., Zhang Q., He J., Wang Q. (2025). Constructing a 3D co-culture in vitro synovial tissue model for rheumatoid arthritis research. Mater. Today Bio.

[69] Meng C., Xia Q., Wu H., Huang H., Liu H., Li Y., Zhang F., Song W. (2020). Photobiomodulation with 630-nm LED radiation inhibits the proliferation of human synoviocyte MH7A cells possibly via TRPV4/PI3K/AKT/mTOR signaling pathway. Lasers Med. Sci..

[70] Modak D., Bhattacharjee S. (2022). A review of fibroblast-like synoviocytes in the pathogenesis of Rheumatoid arthritis: Their activation and the inhibition of their apoptosis. Biomed. Res. Ther..

[71] Ibanez K.R., Huang T.T., Lee J.M. (2024). Combination Therapy Approach to Overcome the Resistance to PI3K Pathway Inhibitors in Gynecological Cancers. Cells.

[72] Kuuliala K., Kuuliala A., Hämäläinen M., Koivuniemi R., Kautiainen H., Moilanen E., Repo H., Leirisalo-Repo M. (2017). Impaired Akt Phosphorylation in Monocytes of Patients with Rheumatoid Arthritis. Scand. J. Immunol..

[73] Ji M., Ryu H.J., Hong J.H. (2021). Signalling and putative therapeutic molecules on the regulation of synoviocyte signalling in rheumatoid arthritis. Bone Jt. Res..

[74] Xie Y., Shi X., Gu K., Han G., Li W., Zhao Q., Jiang B., Feng J., Li J., Gu Y. (2018). PI3K/Akt signaling transduction pathway, erythropoiesis and glycolysis in hypoxia (Review). Int. J. Mol. Med..

[75] Czarnecka K.H., Szmyd B., Barańska M., Kaszkowiak M., Kordiak J., Antczak A., Pastuszak-Lewandoska D., Brzeziańska-Lasota E. (2019). A Strong Decrease in TIMP3 Expression Mediated by the Presence of miR-17 and 20a Enables Extracellular Matrix Remodeling in the NSCLC Lesion Surroundings. Front. Oncol..

[76] Weyand C.M., Goronzy J.J. (2021). The immunology of rheumatoid arthritis. Nat. Immunol..

[77] Wu X., Xu Y., Liang Q., Yang X., Huang J., Wang J., Zhang H., Shi J. (2022). Recent Advances in Dual PI3K/mTOR Inhibitors for Tumour Treatment. Front. Pharmacol..

[78] Wu T.-T., Guo Q.-Q., Chen Z.-L., Wang L.-L., Du Y., Chen R., Mao Y.-H., Yang S.-G., Huang J., Wang J.-T. (2020). Design, synthesis and bioevaluation of novel substituted triazines as potential dual PI3K/mTOR inhibitors. Eur. J. Med. Chem..

[79] Malik B.R., Maddison D.C., Smith G.A., Peters O.M. (2019). Autophagic and Endo-Lysosomal Dysfunction in Neurodegenerative Disease. Mol. Brain.

[80] Petricevic M., Biocina B., Lekic A., Gabelica R. (2014). Antiplatelet therapy at the time of coronary artery surgery: Can a personalized approach improve outcomes?. Eur. J. Cardio-Thorac. Surg..

[81] Sharma S.D., Bluett J. (2024). Towards Personalized Medicine in Rheumatoid Arthritis. Open Access Rheumatol. Res. Rev..

[82] Bao J., Chen Z., Xu L., Wu L., Xiong Y. (2020). Rapamycin protects chondrocytes against IL-18-induced apoptosis and ameliorates rat osteoarthritis. Aging.

[83] Dhanabalan K.M., Dravid A.A., Agarwal S., Sharath R.K., Padmanabhan A.K., Agarwal R. (2022). Intra-articular injection of rapamycin microparticles prevent senescence and effectively treat osteoarthritis. Bioeng. Transl. Med..

[84] Hu Z., Li Y., Zhang L., Jiang Y., Long C., Yang Q., Yang M. (2024). Metabolic changes in fibroblast-like synoviocytes in rheumatoid arthritis: State of the art review. Front. Immunol..

[85] Glaviano A., Foo A.S.C., Lam H.Y., Yap K.C.H., Jacot W., Jones R.H., Eng H., Nair M.G., Makvandi P., Geoerger B. (2023). PI3K/AKT/mTOR signaling transduction pathway and targeted therapies in cancer. Mol. Cancer.

[86] Philippon E.M.L., van Rooijen L.J.E., Khodadust F., van Hamburg J.P., van der Laken C.J., Tas S.W. (2023). A novel 3D spheroid model of rheumatoid arthritis synovial tissue incorporating fibroblasts, endothelial cells, and macrophages. Front. Immunol..

[87] Xia X., He C., Xue Z., Wang Y., Qin Y., Ren Z., Huang Y., Luo H., Chen H.N., Zhang W.H. (2025). Single cell immunoprofile of synovial fluid in rheumatoid arthritis with TNF/JAK inhibitor treatment. Nat. Commun..

[88] Domb A.J., Sharifzadeh G., Nahum V., Hosseinkhani H. (2021). Safety Evaluation of Nanotechnology Products. Pharmaceutics.

[89] Jourdain A.A., Begg B.E., Mick E., Shah H., Calvo S.E., Skinner O.S., Sharma R., Blue S.M., Yeo G.W., Burge C.B. (2021). Loss of LUC7L2 and U1 snRNP subunits shifts energy metabolism from glycolysis to OXPHOS. Mol. Cell.

[90] Rossetti S., Broege A., Sen A., Khan S., MacNeil I., Molden J., Kopher R., Schulz S., Laing L. (2024). Gedatolisib shows superior potency and efficacy versus single-node PI3K/AKT/mTOR inhibitors in breast cancer models. npj Breast Cancer.

[91] Li S., Lin J., Wang C., Liu J., Wang Y., Chen Y., Zhou D. (2025). Synergistic metabolic modulation of fibroblast-like synoviocytes via targeted dual prodrug nanoparticles to mitigate rheumatoid arthritis. Acta Pharm. Sin. B.

[92] European Medicines Agency (2018). Guideline on Strategies to Identify and Mitigate Risks for First-in-Human and Early Clinical Trials With Investigational Medicinal Products.

[93] U.S. Food and Drug Administration (2014). Guidance for Industry: Expedited Programs for Serious Conditions—Drugs and Biologics.

[94] FDA-NIH Biomarker Working Group (2016). BEST (Biomarkers, EndpointS, and Other Tools) Resource [Internet].

