# Supplementary material for: Pharmacological Inhibition of the PI3K/AKT/mTOR Pathway in Rheumatoid Arthritis Synoviocytes: A Systematic Review and Meta-Analysis (Preclinical)
Source: Pharmaceuticals (Basel). 2025 Aug 2;18(8):1152. doi: 10.3390/ph18081152 (PMC12388949; doi:10.3390/ph18081152)
Supplement: Supplementary file 1 [file pharmaceuticals-18-01152-s001.zip › pharmaceuticals-3779354-supplementary/Supplementary/Table S1. PRISMA Chek-list.pdf]

Section and Topic	Item #	Checklist Item	Location in Manuscript
**TITLE**			
Title	1	Identify the report as a systematic review.	Title page: "Effects of PI3K/mTOR Inhibitors on the Functional Properties of Fibroblast-like Synoviocytes in Rheumatoid Arthritis: A Systematic Review and Meta-analysis of in vitro Studies"
**ABSTRACT**			
Abstract	2	See the PRISMA 2020 for Abstracts checklist.	Abstract (Background/Objectives; Methods; Results; Conclusions)
**INTRODUCTION**			
Rationale	3	Describe the rationale for the review in the context of existing knowledge.	§ 1 Introduction, pp. 1–2
Objectives	4	Provide an explicit statement of the objective(s) or question(s) the review addresses.	§ 1 end of Introduction
**METHODS**			
Eligibility criteria	5	Specify the inclusion and exclusion criteria for the review and how studies were grouped for the syntheses.	§ 2.3 "Eligibility criteria" + Supplementary Table S3
Information sources	6	Specify all databases, registers, websites, organisations, reference lists and other sources searched or consulted to identify studies, and the date last searched.	§ 2.2 "Search strategy": PubMed, Europe PMC, Cochrane Library, bioRxiv, medRxiv; last searched May 18, 2025
Search strategy	7	Present the full search strategies for all databases, registers and websites, including any filters and limits used.	§ 2.2 Textbox 1 + Supplementary Table S2
Selection process	8	Specify methods used to decide whether a study met the inclusion criteria, including number of reviewers, independence, conflict resolution.	§ 2.4 "Study selection" + Figure 2 (PRISMA flow; two reviewers, Cohen's $\kappa = 0.92$)
Data collection process	9	Specify methods used to collect data from reports, including number of reviewers, independence, and any automation tools.	§ 2.4 description of standardized extraction form, dual extraction, WebPlotDigitizer
Data items – outcomes	10 a	List and define all outcomes for which data were sought, specifying whether all compatible results were sought.	§§ 2.4, 2.6: proliferation, apoptosis, migration, cytokines, p-AKT/p-mTOR
Data items – other variables	10 b	List and define all other variables for which data were sought (e.g. model characteristics), and describe assumptions about missing or unclear information.	§ 2.3 + Supplementary Table S3: cell models, stimulation parameters, compound characteristics
Study risk of bias assessment	11	Specify methods to assess risk of bias in included studies: tool(s), number of reviewers, independence.	§ 2.5 "Risk of bias assessment" (ToxRTool, two reviewers) + Figure 3 + Supplementary Table S4
Effect measures	12	Specify for each outcome the effect measure(s) used (e.g. Hedges' g).	§ 2.6 "Statistical analysis": SMD (Hedges' g) with 95 % CI
Synthesis methods – eligibility decision	13 a	Describe processes used to decide which studies were eligible for each synthesis (e.g. grouping by outcome).	§ 2.6; groupings detailed in Results §§ 3.1.2–3.1.7
Synthesis methods – data preparation	13 b	Describe methods required to prepare data for presentation or synthesis (e.g. SEM→SD conversions).	§ 2.6 (SEM to SD formula, log-transformation for meta-regression)
Synthesis methods – tabulation/visualization	13 c	Describe methods used to tabulate or visually display results (tables, forest plots).	§ 2.6; Figures 4–7, Tables 1, 3, 4
Synthesis methods – meta-analysis details	13 d	Describe methods used to synthesize results and rationale: model(s), heterogeneity metrics, software.	§ 2.6: random-effects (Sidik–Jonkman), heterogeneity (I^2, Q, τ^2), Python v3.12, R v4.5
Synthesis methods – explore heterogeneity	13 e	Describe methods to explore causes of heterogeneity (subgroup analysis, meta-regression).	§§ 2.6; Results §§ 3.1.5–3.1.6; Tables 1–2; Figures 7–8
Synthesis methods – sensitivity analyses	13 f	Describe sensitivity analyses conducted to assess robustness (e.g. leave-one-out).	§ 2.6; leave-one-out sensitivity in Results and Supplementary
Reporting bias assessment (methods)	14	Describe methods used to assess risk of bias due to missing results (publication bias).	§ 2.6 + § 5.2 qualitative discussion (k<10, reasons for not performing funnel plot/Egger test)

Certainty assessment (methods)	15	Describe methods used to assess certainty (or confidence) in the body of evidence for an outcome.	§ 2.5: "Certainty assessment not performed— all included studies are in vitro; GRADE not applicable."
**RESULTS**			
Study selection – numbers	16 a	Describe results of search and selection process, from records identified to studies included, ideally using a flow diagram.	§ 2.4 + Figure 2 (PRISMA flow diagram)
Study selection – excluded studies	16 b	Cite studies that might meet inclusion criteria but were excluded, and explain why.	§ 2.4: "Reasons for exclusion after full-text review are listed in Supplementary Table S3."
Study characteristics	17	Cite each included study and present its characteristics.	Results § 3.2.1 + Table 3 + Figure 9
Risk of bias in studies (results)	18	Present assessments of risk of bias for each included study.	Results § 3.1.1 + Figure 3
Results of individual studies	19	For all outcomes, present for each study summary statistics and effect estimates with precision (CI), using tables or plots.	Results §§ 3.1.2–3.1.4 + Figures 4–6 + Table 1
Results of syntheses – summary	20 a	For each synthesis, briefly summarise characteristics and risk of bias among contributing studies.	Results § 3.1 introduction to Table 1; RoB summary § 3.1.1
Results of syntheses – statistical	20 b	Present results of all statistical syntheses: summary estimates, precision, heterogeneity, direction of effect.	Results Table 1 + Figures 4–6
Results of syntheses – heterogeneity	20 c	Present results of investigations of possible causes of heterogeneity.	Results §§ 3.1.5–3.1.6; Tables 1–2; Figures 7–8
Results of syntheses – sensitivity	20 d	Present results of all sensitivity analyses.	Leave-one-out sensitivity described in Results § 3.1 and Supplementary
Reporting biases (results)	21	Present assessments of risk of bias due to missing results in a synthesis.	Discussion § 5.2 qualitative assessment of publication bias
Certainty of evidence (results)	22	Present assessments of certainty (or confidence) in the body of evidence for each outcome assessed.	Footnote to Table 1 / summary figures: "Certainty not assessed (all studies in vitro; GRADE not applicable)."
**DISCUSSION**			
Discussion – interpretation	23 a	Provide a general interpretation of the results in the context of other evidence.	Discussion §§ 4.1–4.3
Discussion – limitations of evidence	23 b	Discuss any limitations of the evidence included in the review.	Discussion § 5.1
Discussion – limitations of review process	23 c	Discuss any limitations of the review processes used.	Discussion § 5.2
Discussion – implications	23 d	Discuss implications of the results for practice, policy, and future research.	Discussion §§ 4.4–4.7
**OTHER INFORMATION**			
Registration and protocol – registry	24 a	Provide registration information for the review, including register name and registration number.	§ 2.1: PROSPERO, ID CRD420251058185
Registration and protocol – access	24 b	Indicate where the review protocol can be accessed.	§ 2.1: PROSPERO online
Registration and protocol – amendments	24 c	Describe and explain any amendments to information provided at registration or in the protocol.	§ 2.1: "No other amendments to the protocol were made."
Support	25	Describe sources of financial or non-financial support for the review, and the role of the funders or sponsors.	Funding statement: National Natural Science Foundation of China (82271822)
Competing interests	26	Declare any competing interests of review authors.	"The authors declare no conflict of interest."
Availability of data, code and materials	27	Report which of the following are publicly available and where they can be found: template data collection forms; data extracted from included studies; data used for analyses; analytic code; any other materials used in the review.	Data Availability section + Supplementary Tables S1–S4 + link to Zenodo/OSF
