# Supplementary material for: Pharmacological Inhibition of the PI3K/AKT/mTOR Pathway in Rheumatoid Arthritis Synoviocytes: A Systematic Review and Meta-Analysis (Preclinical)
Source: Pharmaceuticals (Basel). 2025 Aug 2;18(8):1152. doi: 10.3390/ph18081152 (PMC12388949; doi:10.3390/ph18081152)
Supplement: Supplementary file 1 [file pharmaceuticals-18-01152-s001.zip › pharmaceuticals-3779354-supplementary/Supplementary/Table S2. Search strategy.pdf]

Database	Search Terms	Records Retrieved
**PubMed**	("rheumatoid arthritis"[tiab] OR "Arthritis, Rheumatoid"[MeSH])<br>AND<br>("fibroblast-like synoviocytes"[tiab] OR "FLS"[tiab] OR "synovial fibroblasts"[tiab] OR "Synovial Membrane"[MeSH])<br>AND<br>("PI3K"[tiab] OR "PI3K/AKT"[tiab] OR "AKT"[tiab] OR "mTOR"[tiab] OR "PI3K/mTOR"[tiab] OR "Phosphatidylinositol 3-Kinases"[MeSH] OR "PI-103"[tiab] OR "BEZ235"[tiab] OR "LY294002"[tiab] OR "Wortmannin"[tiab])<br>AND<br>("in vitro"[tiab] OR "cell culture"[tiab])<br>AND<br>(proliferation[tiab] OR migration[tiab] OR invasion[tiab] OR apoptosis[tiab] OR cytokines[tiab])	**76**
**PubMed**	(rheumatoid arthritis OR Arthritis, Rheumatoid)<br>AND<br>(fibroblast-like synoviocytes OR FLS OR synovial fibroblasts OR Synovial Membrane)<br>AND<br>(PI3K OR PI3K/AKT OR AKT OR mTOR OR PI3K/mTOR OR Phosphatidylinositol 3-Kinases OR PI-103 OR BEZ235 OR LY294002 OR Wortmannin)<br>AND<br>(in vitro OR cell culture)<br>AND<br>(proliferation OR migration OR invasion OR apoptosis OR cytokines)	**123**
**PubMed**	(rheumatoid arthritis OR Arthritis, Rheumatoid)<br>AND<br>(fibroblast-like synoviocytes OR FLS OR synovial fibroblasts OR Synovial Membrane)<br>AND<br>(PI3K OR PI3K/AKT OR AKT OR mTOR OR PI3K/mTOR OR Phosphatidylinositol 3-Kinases OR PI-103 OR BEZ235 OR LY294002 OR Wortmannin)<br>AND<br>(in vitro OR cell culture)	**131**
**PubMed**	(rheumatoid arthritis OR Arthritis, Rheumatoid)<br>AND<br>(fibroblast-like synoviocytes OR FLS OR synovial fibroblasts OR Synovial Membrane)<br>AND<br>(PI3K OR PI3K/AKT OR AKT OR mTOR OR PI3K/mTOR OR Phosphatidylinositol 3-Kinases OR PI-103 OR BEZ235 OR LY294002 OR Wortmannin)	**407**
**PubMed**	(rheumatoid arthritis OR RA)<br>AND<br>(synoviocytes OR fibroblasts OR FLS)<br>AND<br>(PI3K OR AKT OR mTOR)	**406**
**PubMed**	("rheumatoid arthritis"[tiab] OR RA[tiab])<br>AND<br>(MH7A[tiab] OR "MH7 A"[tiab])<br>AND<br>(PI3K OR AKT OR mTOR OR "PI3K/AKT" OR "PI3K/mTOR" OR "PI-103" OR BEZ235 OR LY294002 OR Wortmannin)<br>AND<br>("in vitro" OR "cell culture")	**23**

**PubMed**	("rheumatoid arthritis" OR RA)<br>AND<br>(MH7A OR "MH7 A")<br>AND<br>("fibroblast-like synoviocytes" OR synoviocyte OR FLS OR "synovial fibroblasts")<br>AND<br>(PI3K OR AKT OR mTOR OR "PI3K/AKT" OR "PI3K/mTOR" OR "PI-103" OR BEZ235 OR LY294002 OR Wortmannin)<br>AND<br>("in vitro" OR "cell culture")	**12**
**Europe PMC**	("rheumatoid arthritis" OR "Arthritis, Rheumatoid")<br>AND<br>("fibroblast-like synoviocytes" OR FLS OR "synovial fibroblasts" OR "Synovial Membrane")<br>AND<br>(PI3K OR "PI3K/AKT" OR AKT OR mTOR OR "PI3K/mTOR" OR "Phosphatidylinositol 3-Kinases" OR PI-103 OR BEZ235 OR LY294002 OR Wortmannin)<br>AND<br>("in vitro" OR "cell culture")<br>AND<br>(proliferation OR migration OR invasion OR apoptosis OR cytokines)	**1148**
**Europe PMC**	("rheumatoid arthritis" OR RA)<br>AND<br>(MH7A OR "MH7 A")<br>AND<br>(PI3K OR AKT OR mTOR OR "PI3K/AKT" OR "PI3K/mTOR" OR PI-103 OR BEZ235 OR LY294002 OR Wortmannin)<br>AND<br>("in vitro" OR "cell culture")	**210**
**Europe PMC**	("rheumatoid arthritis" OR RA)<br>AND<br>(MH7A OR "MH7 A")<br>AND<br>("fibroblast-like synoviocytes" OR synoviocyte OR FLS OR "synovial fibroblasts")<br>AND<br>(PI3K OR AKT OR mTOR OR "PI3K/AKT" OR "PI3K/mTOR" OR PI-103 OR BEZ235 OR LY294002 OR Wortmannin)<br>AND<br>("in vitro" OR "cell culture")	**174**
**Cochrane Library**	("rheumatoid arthritis" OR RA)<br>AND<br>(synoviocyte* OR "synovial fibroblast*" OR "fibroblast-like synoviocyte*" OR RASF OR RA-FLS OR FLS)<br>AND<br>(PI3K OR "phosphatidylinositol 3-kinase" OR "PI 3-kinase" OR AKT OR "protein kinase B" OR mTOR OR "mechanistic target of rapamycin")	**0**
**Cochrane Library**	("rheumatoid arthritis" OR "RA" OR "synovitis")<br>AND<br>("phosphoinositide 3-kinase" OR "PI3K" OR "PI 3K" OR "PI3 kinase" OR "AKT" OR "protein kinase B" OR "mammalian target of rapamycin" OR "mechanistic target of rapamycin" OR "mTOR" OR "mTORC1" OR "mTORC2")<br>AND<br>("in vitro" OR "cell culture" OR "cell line*" OR "primary cell*" OR "cultured synoviocytes" OR "immortalized cell*")	**0**
