# Supplementary material for: Pharmacological Inhibition of the PI3K/AKT/mTOR Pathway in Rheumatoid Arthritis Synoviocytes: A Systematic Review and Meta-Analysis (Preclinical)
Source: Pharmaceuticals (Basel). 2025 Aug 2;18(8):1152. doi: 10.3390/ph18081152 (PMC12388949; doi:10.3390/ph18081152)
Supplement: Supplementary file 1 [file pharmaceuticals-18-01152-s001.zip › pharmaceuticals-3779354-supplementary/Supplementary/Table S3. Eligibility Criteria and MICRO (Inclusion-Exclusion).pdf]

Element	Inclusion	Exclusion
**Model**	- Primary RA-FLSs isolated from synovial tissue of patients with rheumatoid arthritis	- FLSs obtained from healthy donors
	- Commercial RA-FLS cell lines (e.g., HFLS-RA)	- Animal-derived FLSs
	- Immortalized lines (e.g., MH7A)	- Non-fibroblast cell lines
	- Any cell cultures explicitly stated as derived from RA synovium	- In vivo studies
**Intervention**	- PI3K inhibitors (class-specific, isoform-specific, allosteric)	- Compounds not directly affecting PI3K/AKT/mTOR pathway
	- AKT inhibitors (e.g., MK-2206)	- Combination interventions where the effect of the inhibitor cannot be distinguished
	- mTOR inhibitors (e.g., rapamycin, everolimus)	- Genetic interventions only (siRNA, shRNA) without a pharmacological component
	- Dual PI3K/mTOR inhibitors (e.g., PI-103, BEZ235)	- Agents with primary molecular targets outside the PI3K/AKT/mTOR cascade
	- Other small molecules with direct evidence of targeting the PI3K/AKT/mTOR cascade in RA-FLS in vitro	- Herbal extracts/multicomponent mixtures with unclear specificity
**Comparator**	- Vehicle control (e.g., DMSO, PBS)	- No clearly described control group
	- RA-FLSs stimulated with cytokines (TNF-α, IL-1β, LPS) without inhibitor	- Controls treated with another active agent affecting the PI3K/AKT/mTOR pathway
	- Naïve (untreated) controls	- Controls with different culture conditions compared to intervention group
	- Combinations of vehicle + cytokine	- Controls represented only by the “0 h” time point without parallel incubation
		- Control data taken from other publications or unrelated experiments
**Readout/ Outcomes**	- Proliferation (CCK-8, EdU, Ki-67)	- Descriptive data only
	- Migration/invasion (wound healing, transwell)	- No quantitative assessment
	- Cytokine production (IL-6, IL-8, IL-1β; ELISA, qPCR, WB)	- Outcomes unrelated to PI3K/AKT/mTOR or not involving FLS
	- Expression/phosphorylation of PI3K/AKT/mTOR components (AKT, p-AKT, mTOR, p-mTOR)	
	- MMPs, apoptosis, metabolic and oxidative markers (if quantitatively assessed)	
**Other Criteria**	- Publication years: 2010–2025	- Reviews, case reports, conference abstracts
	- Minimum duration of intervention: 0 weeks/hours (in vitro)	- In vivo/animal studies
	- Minimum follow-up: not applicable	- Studies lacking outcome or control group
	- Original quantitative in vitro studies using pharmacological PI3K/mTOR inhibitors	- Studies with combination interventions may be included only if a separate group with inhibitor only is [illegible]
	- Studies reporting at least one quantitative outcome with an appropriate control group	- Studies presenting graphical data only are included in qualitative analysis but not in meta-analysis
	- Studies using primary, commercial, or immortalized FLS	
	- Minimum: ≥3 biological replicates or ≥3 independent donors for inclusion in meta-analysis	
